# *Chimaeribacter arupi* a new member of the Yersineacea family has the characteristics of a human pathogen

**DOI:** 10.3389/fcimb.2023.1277522

**Published:** 2023-10-06

**Authors:** Matthias Riediger, Katharina Hoffmann, Riekje Isberner, Annika Dreyer, Aljoscha Tersteegen, Pauline Marquardt, Achim J. Kaasch, Andreas E. Zautner

**Affiliations:** ^1^ Institut für Medizinische Mikrobiologie und Krankenhaushygiene, Medizinische Fakultät der Otto-von-Guericke Universität Magdeburg, Magdeburg, Germany; ^2^ Universitätskinderklinik, Medizinische Fakultät der Otto-von-Guericke Universität Magdeburg, Magdeburg, Germany; ^3^ Institut für Medizinische Mikrobiologie und Virologie, Universitätsmedizin Göttingen, Göttingen, Germany; ^4^ Center for Health and Medical Prevention (CHaMP), Center for Health and Medical Prevention, Otto-von-Guericke-University Magdeburg, Magdeburg, Germany

**Keywords:** *Chimaeribacter arupi*, “*Nissabacter archeti*”, genome, comparative genomics, case report, blood stream infection, virulence factors

## Abstract

*Chimaeribacter arupi* (heterotypic synonym: “*Nissabacter archeti*”) is a facultative anaerobic, newly described Gram-negative rod and belongs to the Yersineacea family. Here, we report the case of a 19-month-old female infant patient who presented to the emergency unit with somnolence and fever. *C. arupi* was isolated from a positive blood culture, taken via an implanted Broviac catheter, proving a bloodstream infection by the pathogen. The objective of this study was to utilize whole genome sequencing to assess the genes encoding potential virulence associated factors, which may play a role in host tropism, tissue invasion and the subsequent stages in the pathogenesis of a bloodstream infection with *C. arupi*. The genome of the isolate was completely sequenced employing Illumina MiSeq and Nanopore MinION sequencing and the presumptive virulence associated factors and antimicrobial resistance genes were investigated in more detail. Additionally, we performed metabolic profiling and susceptibility testing by microdilution. The presence of predicted TcfC-like α-Pili suggests that *C. arupi* is highly adapted to humans as a host. It utilizes flagellar and type IV pili-mediated motility, as well as a number of γ_1_-pili and a σ-pilus, which may be used to facilitate biofilm formation and adherence to host epithelia. Additionally, long polar fimbriae may aid in tissue invasion. The bacterium possesses antioxidant factors, which may enable temporary survival in phagolysosomes, and a capsule that potentially provides protection from phagocytosis. It may acquire iron ions from erythrocytes through the type 6 secretion system and hemolysins. Furthermore, the isolate exhibits beta-lactamase-mediated penicillin and aminopenicillin resistance. Based on the analysis of the whole genome, we conclude that *C. arupi* possesses virulence factors associated with tissue invasion and may thus be a potential opportunistic pathogen of bloodstream infections.

## 1 Introduction

A 19-months-old female infant presented to our emergency department with sleepiness and fever (39.6°C) since the morning of the admission day in August 2022. The patient was a former premature baby born in January 2021 in the 35th week of gestation (birth weight 2,490 g). She suffered from a congenital gastroschisis and a volvulus. Additionally, a meconium ileus led to cystic meconium peritonitis in the perinatal period. On her first day of life, the surgeons had to resect parts of the jejunum, ileum, ascending colon with caecum and the right colic flexure resulting in a short bowel syndrome in the later life. The patient was provided with an implanted Broviac catheter due to her short bowel syndrome. The patient was well known to our hospital due to recurrent Broviac infections and defects, which led to septicemia and required multiple Broviac replacements in August 2021, February 2022, April 2022, and July 2022. Additionally, the patient experienced recurrent mechanical ileus in June 2021, December 2021, January 2022, February 2022, and March 2022, which was treated with erythromycin. Prior to the current case, the patient had been hospitalized 13 times, including the stay in the neonatal intensive care unit. Developing a comprehensive diet plan has been challenging due to short bowel syndrome. In addition to total parenteral nutrition (TPN), high-calorie nutrition has been necessary. However, there have been difficulties with the acceptance of high-calorie foods, and a low tolerance for them, resulting in slow growth and weight gain. After jejunostomy a colojejunal re-anastomosis was performed without any complications. In September 2021, a colonoscopy with biopsy revealed the initial histological findings of Crohn's disease.

During the hospital stay in August 2022, special nutrition, Neocate^®^ Infant Formula, was well tolerated besides total parenteral nutrition (TPN). There were no clinical signs indicating an infection in the surrounding area of the Broviac catheter. The mother reported that antipyretic suppositories worked well. The patient passed 4 to 5 times stool per day. However, inconspicuous clinical examination and the irritation-free insertion site did not definitively rule out the possibility of septicemia. There was still a chance of recurrent bloodstream infection originating from the implanted Broviac catheter. As a consequence, blood cultures were collected and additional stool samples were obtained.

Here we present a clinical case involving a 19-month-old toddler with a bloodstream infection caused by *C. arupi*. We sequenced the genome of the isolate and analyzed its genomic features that may be associated with virulence, host tropism, and tissue invasion. To the best of our knowledge, with *C. arupi* DSM115072 we describe the first complete genome sequence of this microbial species.

## 2 Background

### 2.1 The genus *Chimaeribacter* (“*Nissabacter*”)

The genus *Chimaeribacter* with the three microbial species *Chimaeribacter arupi*, *Chimaeribacter coloradensis*, and *Chimaeribacter californicus* was first described and validly published in 2020 by Rossi and Fisher employed by the Associated Regional and University Pathologists (ARUP) Laboratories in Salt Lake City, Utah, USA (Rossi and Fisher, 2020). The genus designation was chosen because of the multiple characteristics typical for distinct Enterobacterales genera exhibited within a single organism. In 2017, however, the genus “*Nissabacter*” with the only species “*Nissabacter archeti*” was described for the first time but not validly published (Mlaga et al., 2017). The genus and species designation, *“Nissabacter archeti*”, was chosen because the first isolate was isolated from a scalp pustule of a 29 year old man treated in the Archet 2 Hospital in Nice, France (Mlaga et al., 2017). The classification of the isolate from Nice as a new microbial species was based on an unclosed genome sequence consisting of 50 contigs (BioSample ID: SAMEA17980918). About two years later, the first closed chromosome sequence of a “*Nissabacter*” sp. isolate was published (Gaultier et al., 2019). This isolate, named SGAir0207, originated from an air sample from Singapore and belongs to another so far unspecified microbial species of the genus “*Nissabacter*”, which is different from the microbial species “*Nissabacter archeti*” (Gaultier et al., 2019). The scaffold genome sequence of a second *“Nissabacter archeti*” isolate (JGM97) cultured from a *Drosophila* sp. caught in a kitchen was published in 2021 (BioSample ID: SAMN17146139). Rossi and Fisher showed that the microbial species *Chimaeribacter arupi* and “*Nissabacter archeti*” were identical, thus “*Nissabacter archeti*” is a heterotypic synonym for *Chimaeribacter arupi*. Therefore, based on the valid publication of the genus *Chimaeribacter* and the non-valid publication of the genus “*Nissabacter*”, preference should be given to the genus name *Chimaeribacter* and the genus name “*Nissabacter*” should be abandoned. The genus *Chimaeribacter* (“*Nissabacter*”) belongs to the order Enterobactereales and the family Yersiniaceae, which also includes the previously described Genera *Rahnella*, *Ewingella*, *Rouxiella*, *Yersinia, Serratia*, *Chania*, and *Samsonia* (Sutra et al., 2001; Rossi and Fisher, 2020; Soutar and Stavrinides, 2020).

### 2.2 Culture and morphology

**Figure 1:**
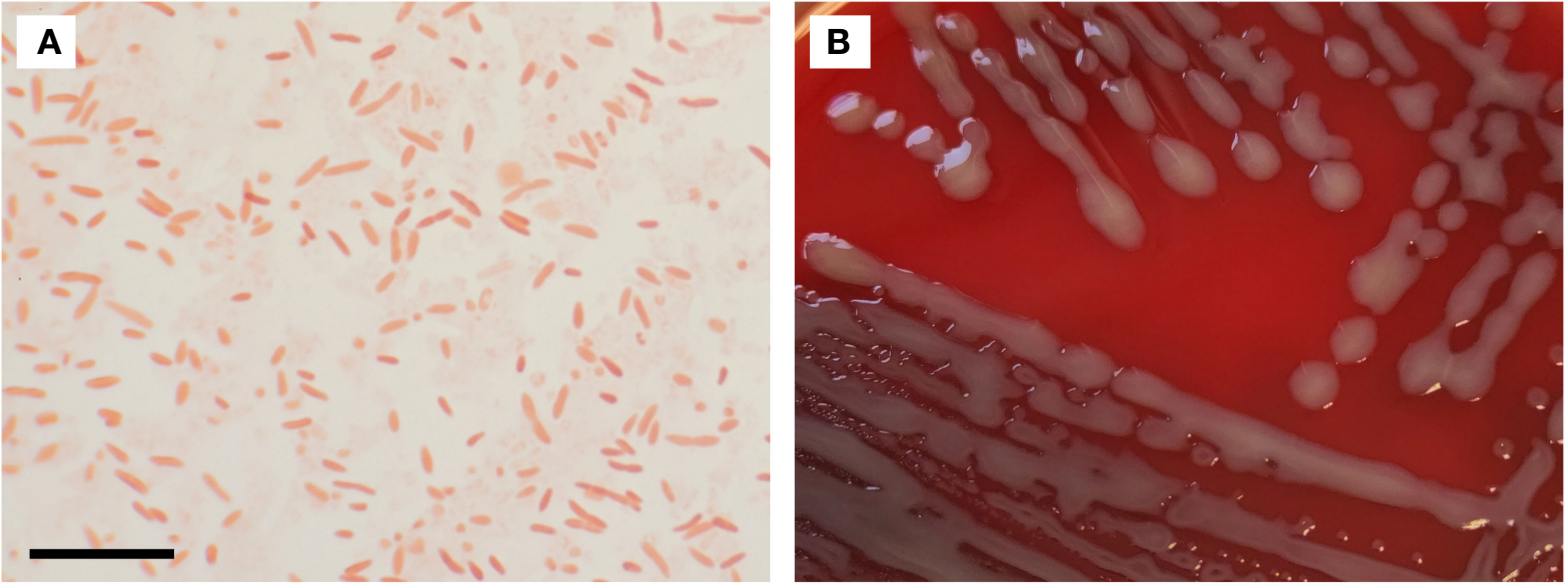
**(A)** Gram stain of *Chimaeribacter arupi*. The scale bar in the lower left corner corresponds to 10 µm. Gram-negative very pleomorphic rods are observable. **(B)** Depicted are *Chimaeribacter arupi* colonies grown on Columbia agar supplemented with sheep blood grown overnight at 36°C. Bacterial colonies are relatively large, mucoid, gray in color with a light yellowish tinge.

In the Gram stain *Chimaeribacter arupi* grown at 22°C or 35°C under aerobic atmospheric conditions presents as Gram-negative pleomorphic, non-spore-forming rods with a single cell length of 0.9 µm to 4.6 µm and a width ranging from 0.4 µm to 1.0 µm (**Figure 1A**). The strong variation in the size of individual bacterial cells is characteristic (Rossi and Fisher, 2020). In terms of culture conditions, *Chimaeribacter arupi* can be cultivated under aerobic and anaerobic conditions on Columbia sheep blood or chocolate blood agar. When grown on Columbia agar, the colonies are smooth white-greyish and circular with a diameter of approximately 2 to 3 mm after overnight incubation (ca. 12 h). With longer incubation time, the colonies become slightly yellowish, show weak hemolysis, and appear mucoid (**Figure 1B**).

### 2.3 Genomic and metabolic features

To date, there are no complete genomes (closed chromosome) accessible for *C. arupi*. Based on the available contigs *C. arupi* has a genome size of about 4.7-5.0 Mb and a G+C content of 58.5-59.8%. The accessible genomes contain 4,124-4,481 coding genes, 1-3 CRISPR arrays as well as one prophage sequence. A carotenoid synthesis operon was described, which is probably responsible for yellow pigmentation of the colonies, analogous to some Enterobacterales with yellow pigmented colonies (Rossi and Fisher, 2020). Rossi and Fisher conducted metabolic analyses on different *Chimaeribacter* isolates and found that they can utilize citrate, reduce nitrate, and hydrolyze aesculin. Almost all isolates showed gelatinase activity, similar to the closely related genus *Serratia*, and the ability to ferment various sugars and carbohydrates. However, they were unable to produce H_2_S, hydrolyze urea, produce indole, or form acetoin. They also lacked certain biochemical activities and were unable to ferment several other substances. Overall, the biochemical studies showed the greatest similarity in biochemical and fermentation patterns with some species of the genus *Serratia* and *Proteus*. The gas chromatographic analysis of the fatty acid profiles by Rossi and Fisher showed a similar composition within the different *Chimaeribacter* isolates. In particular, C16: 0; C17: 0 cyclo; C14: 0 3-OH/iso-C16: 1 I; C18: 1 ω7c; and C 16: 1 ω7 c/C 16: 1 ω6 c were detected. Overall, the fatty acid profile studied showed highest similarity to members of the genera *Yersinia*, *Proteus* and *Alcaligenes*, among others (Rossi and Fisher, 2020).

## 3 Methods

### 3.1 Culture, species identification, metabolic profiling, and susceptibility testing

In accordance with the diagnostic routine, a blood sample was drawn and a BD BACTEC^™^ Peds Plus^™^ blood culture bottle was inoculated, and consecutively placed in the intended BACTEC FX incubator (Becton Dickinson, Franklin Lakes, New Jersey, USA). Positive blood cultures were plated on Columbia agar supplemented with 5% sheep blood (aerobic), chocolate-agar (aerobic), and CHROMagar KPC/ESBL (aerobic). Gram stains were prepared using a PREVI^®^ Color Gram V2 staining machine (bioMérieux, Marcy-l’Étoile, France).

Microbial species identification by MALDI-TOF mass spectrometry using a Vitek MS or biochemical methods such as a VITEK^®^ 2 GN ID card used in a VITEK^®^ 2 XL device (bioMérieux, Marcy-l’Étoile, France) was attempted. Furthermore, the 16S rRNA gene was amplified (Kommedal et al., 2009) and Sanger sequencing of the amplicon was performed employing the ExoSAP-IT™ and BigDye Terminator v3.1 Cycle Sequencing kit (Applied Biosystems by Thermo Fisher Scientific, Vilnius, Lithuania.) on the ABI PRISM 3130xl Genetic Analyzer (Applied Biosystems, Foster City, USA).

Metabolic profiling, especially carbohydrate fermentation, was assessed using API 20E and API 50CH strips (bioMérieux, Marcy-l’Étoile, France) according to the manufacturer’s instructions. The color changes of the indicator reactions were read and documented after 24 h and 48 h incubation at 37°C.

Susceptibility testing was performed using the MICRONAUT IVD antimicrobial susceptibility testing (AST) system in combination with the minimum inhibitory concentration (MIC) plate MCN-S MH Hannover GN3 (Sifin diagnostics Gmbh, Berlin, Germany) according to the manufacturer’s instructions.

### 3.2 DNA extraction, library preparation and whole genome sequencing

Isolation of bacterial DNA was performed via the cetyltrimethylammonium bromide (CTAB) method as described previously by Bechman et al. (Bechmann et al., 2022). Library preparation for Illumina paired-end sequencing was performed using the NEBNext^®^ Ultra™ II FS DNA Library Prep Kit for Illumina #E6177 (New England Biolabs GmbH, Frankfurt am Main, Germany). Libraries were barcoded using the NEBNext® Multiplex Oligos for Illumina^®^ 96 Unique Dual Index Primer Pairs #E6440S/L (New England Biolabs GmbH, Frankfurt am Main, Germany) and sequenced using the MiSeq Reagent Kit v2 (500-cycles, Illumina) as described by the manufacturer. Barcoded libraries for Oxford Nanopore long-read sequencing were prepared using the Rapid Barcoding Kit 96 (SQK-RBK110.96) according to the manufacturer’s instructions and sequenced on a R9.4.1 flow cell (FLO-MIN106) on the MinION platform (Oxford Nanopore technologies ltd., Oxford, United Kingdom).

### 3.3 Genome assembly and quality control

Illumina paired-end reads were preprocessed using fastp (https://github.com/OpenGene/fastp, v0.23.2), and filtlong (*parameters:* –min_length 1000 –keep_percent 95, https://github.com/rrwick/Filtlong, v0.2.1) was used for long reads. Genomes were assembled using a long read consensus assembly approach presented in the trycycler software package (https://github.com/rrwick/Trycycler, v0.5.3). In short, long reads were sampled into 20 unique subsets of reads with a 100x sequencing depth and each sets of 4 subsets were independently assembled using *canu* v1.8.1 (Koren et al., 2017), *flye* v2.9.1-b1780 (Kolmogorov et al., 2019), miniasm *v0.3 (* https://github.com/lh3/miniasm *)*, *minipolish* v0.1.3 (Wick and Holt, 2019), *necat* v0.0.1 (https://github.com/xiaochuanle/NECAT) and *raven* v1.8.1 (https://github.com/lbcb-sci/raven) to generate 20 independent long-read assemblies. Subsequently, the trycycler functions cluster, reconcile, msa, partition, and consensus were applied on the set of assemblies for each sample to generated consensus assemblies. Finally, the consensus assemblies were polished using the long read polisher medaka v1.7.2 (https://github.com/nanoporetech/medaka) and short read polisher *polypolish* v0.5.0 (https://github.com/rrwick/Polypolish) (Wick and Holt, 2022). The assembly quality was assessed using QUAST v5.2.0 (Gurevich et al., 2013).

### 3.4 Annotations and further bioinformatic analyses

The assembly was annotated using the NCBI Prokaryotic Genome Annotation Pipeline (PGAP) stand-alone software version 2022-12-13.build6494 (Tatusova et al., 2016; Haft et al., 2018; Li et al., 2021). A taxonomy check was performed using fastANI v1.33, integrated in the PGAP software. The full-length 16S rDNA sequence was additionally analyzed using BLASTn search against NCBI 16S ribosomal RNA sequence database. The hits were aligned using clustalW v2.1, phylogentic tree was generated using IQ-TREE v2.2.2.7 (Minh et al., 2020) and visualized using Figtree v1.4.4 (https://github.com/rambaut/figtree). Core genome analysis was performed using Panaroo v1.3.3 (Tonkin-Hill et al., 2020). Functional annotation via determination and categorization of orthologous groups (OGs) was performed using EggNOG 5.0 (Huerta-Cepas et al., 2019), eggNOG-mapper v2 (Cantalapiedra et al., 2021), and MGCplotter v1.0.1 (https://github.com/moshi4/MGCplotter). Pie charts were generated using matplotlib in python 3 (van Rossum and Drake, 2009). Screening for the presence of antimicrobial resistance genes and point mutations that cause antimicrobial resistance was conducted using three tools: Resfinder V4.1 (Zankari et al., 2012), PointFinder (Zankari et al., 2017), and ResFinderFG V1.0 (Sommer et al., 2009; Pehrsson et al., 2016). The presence of *C. arupi* in the human intestinal microbiome was checked by a BLAST search against the human gut metagenome 16S ribosomal RNA database available at https://www.ncbi.nlm.nih.gov/Taxonomy/Browser/wwwtax.cgi?id=408170.

## 4 Results and discussion

### 4.1 Microbiological results

MALDI-TOF MS and VITEK^®^ 2 GN ID card based analyses did not produce meaningful results for species identification. 16S rRNA gene sequence analysis revealed a 632 bp PCR product that displayed a 99.51% sequence identity to “*Nissabacter*” sp. SGAir0207 and 99.03% to “*Nissabacter archeti*” strain 2134. Additionally, it exhibited a similar 99.03% sequence identity to *Chimaeribacter arupi* strain 2016-Iso3, with a query coverage of 97%, according to a BLAST search conducted on the NCBI GenBank database. Consequently, our bacterial isolate was identified as *Chimaeribacter arupi*. The isolate was sent to the Leibniz Institute DSMZ - German Collection of Microorganisms and Cell Cultures GmbH, was confirmed as *C. arupi* and assigned the identifier DSM115072.

To further confirm the species identity, we compared the metabolic profile of the isolated bacterium using API E, API CH50, and VITEK^®^ 2 GN ID card with the published metabolic characteristics of *C. arupi*. Rossi and Fischer determined the metabolic profiles of 4 *C. arupi* isolates and 5 other isolates from the genus *Chimaeribacter* belonging to other species using API E and API CH50 (Rossi and Fisher, 2020). Compared to the four *C. arupi* isolates, the metabolic profile of our isolate matches in most parameters (**Supplementary Table 1**). There were only differences observed in 14 parameters, which are commonly variable even among the metabolic profiles of the four previously published isolates. Fermentation of D-lactose, D-raffinose and potassium-2-ketogluconate showed a weak reaction in the APIs while showing a positive reaction for the four published isolates. Fermentation of starch (amidon) showed a weak reaction in the APIs besides a negative reaction in the four published isolates. Details are listed in **Table 1**. Additionally, metabolic parameters determined using a Vitek2 GN ID card were also given in **Table 1**.

**Table 1:** Metabolic characteristics of *C. arupi* DSM115072 that may be considered aberrant or variable compared to published metabolic characteristics of other *C. arupi* isolates (Rossi and Fisher, 2020).

	characteristic	*C. arupi* DSM115072	*C. arupi* 2016-Iso1	*C. arupi* 2016-Iso2	*C. arupi* 2016-Iso3	*C. arupi* 2013-Iso5
API50CH	fermentation of:					
	glycerol	(+)	+	–	–	+
	D-arabinose	(+)	–	–	–	+
	L-arabinose	+	+	–	+	+
	dulcitol	+	+	–	+	+
	amygdalin	-/+^d^	–	–	+	+
	D-lactose	(+)	+	+	+	+
	sucrose	- ^c v^	–	+	+	+
	D-raffinose	(+)	+	+	+	+
	starch (amidon)	(+)	–	–	–	–
	potassium-gluconate	–	+	–	–	–
	potassium-2-ketogluconate	(+)	+	+	+	+
API 20E						
	β-galactosidase (ONPG)	-/+^w^	–	–	–	–
	citrate utilization	(+)/+^w^	+	+	+	+
	gelatinase	(+)	+	+	+	–
Vitek2 GN ID						
	Ala-Phe-Pro-arylamidase	–				
	L-pyrrolydonyl-arylamidase	+				
	glutamyl-arylamidase-pNA	–				
	gamma-glutamyl-transferase	–				
	beta-glucosidase	+				
	beta-xylosidase	–				
	beta-alanin-arylamidase-pNA	–				
	L-prolin-arylamidase	–				
	lipase	–				
	palatinose	–				
	tyrosin-arylamidase	–				
	malonat	–				
	L-lactate alkalization	–				
	alpha-glucosidase	–				
	succinate-alkalization	–				
	beta-N-acetyl-galactosaminidase	+				
	phosphatase	–				
	glycin-arylamidase	–				
	L-histidine assimilation	–				
	coumarate	–				
	beta-glucuronidase	–				
	O/129-resistance (comp.vibrio.)	+				
	Glu-Gly-Arg-arylamidase	–				
	L-malate Assimilation	–				
	Ellman’s reagent	–				
	L-lactat-assimilation	–				

^c^ consistent results in API50CH and API20E, ^d^ divergent results in API50CH and API20E, ^v^ consistent results in API50CH/API20E and Vitek2 GN ID card, ^w^ divergent results in API50CH/API20E and Vitek2 GN ID card.

Antimicrobial susceptibility testing (AST) results determined retrospectively using the MICRONAUT IVD AST system by microdilution revealed the minimum inhibitory concentrations (MIC) listed in **Table 2**. Since *C. arupi* belongs to the family Yersineacea and thus to the order Enterobacterales, the corresponding Enterobacterales breakpoints according to the EUCAST V12.0 (European Committee on Antimicrobial Susceptibility Testing) guidelines as well as the CLSI M100S, 30^th^ ed. (U.S. Clinical Laboratory Standards Institute) breakpoints for Enterobacteriaceae, were used for MIC interpretation. According to the test results, there was resistance to aminopenicillins (at least limited efficacy according to CLSI) and thus also to unmodified penicillins. In contrast, there was susceptibility to acylureidopenicillins, cephalosporins, carbapenems, fluoroquinolones, trimethoprim-sulfamethoxazol, colistin, tigecycline and aminoglycosides.

**Table 2:** Antimicrobial susceptibility testing results of *Chimaeribacter arupi* DSM115072 to various antimicrobial substances and interpretation according to EUCAST Enterobactereales breakpoints as well as CLSI breakpoints for Enterobacteriaceae.

antimicrobial substance	MIC [mg/L]	susceptibility EUCAST Enterobacterales	susceptibility CLSI *Enterobacteriaceae*
ampicillin	=16	R	I
ampicillin-sulbactam	≤0.5/4	S	S
piperacillin/tazobactam	=0.5/4	S	S
cefuroxime	=2	–	S
cefotaxime	≤0.125	S	S
ceftazidime	≤0.5	S	S
imipenem	≤1	S	S
meropenem	≤0.125	S	S
ertapenem	≤0.125	S	S
ciprofloxacin	≤0.0625	S	S
moxifloxacin	≤0.0625	S	–
trimethoprim-sulfamethoxazol	≤0.25/4.75	S	S
fosfomycin	>128	–	–
colistin	≤0.5	S	–
tigecycline	≤0.25	–	–
gentamicin	≤0.5	S	S
tobramycin	≤0.5	S	S

### 4.2 Clinical course

Due to suspected Broviac catheter associated septicaemia a calculated intravenous antibiotic therapy with meropenem (60 mg/kg/d divided into 3 doses = 150 mg every 8 h) and vancomycin (50 mg/kg/d divided into 4 doses = 100 mg every 6 h) was started. Additionally, antipyretic therapy was prescribed (paracetamol and metamizole each up to 4 x 100 mg/d). Long-term-medication with the administration of mesalazine (3 x 150 mg/d p.o.), NaHCO_3_ 8,4% (6 x 2,5 mL/d p.o.), enoxaparin-natrium (1 x 5 mg/d s.c.), and erythromycin (3 x 20 mg/d p.o., as permanent therapy due to recidivating mechanical ileus) was continued. Stool samples were unsuspicious concerning bacterial or viral infection. When the blood cultures showed a bloodstream infection with *C. arupi*, we discontinued the use of vancomycin based on the Gram stain results. We continued treatment with meropenem based on the results of antimicrobial susceptibility testing (significant lower MIC-value in comparison to ceftazidim). Meropenem was continued for a total of 14 days to eliminate any potential biofilm formation on the surface of the implanted Broviac catheter, with the aim of preserving its functionality. Under therapy, the fever (max. 39.6°C on admission day) stopped and infectious disease markers (CRP max. 96 mg/L) decreased. Repeated blood culture during the patient’s stay showed no additional pathological finding, so we decided against a removal of the Broviac catheter. Supportively a persistent diaper dermatitis was treated with laser therapy. After seventeen days, the patient was discharged from hospital (**Table 3**).

**Table 3:** Disease progression timeline.

day	symptoms, signs, medical findings, procedures
1	presentation with fever and sleepiness, drawing of blood cultures, CRP (65 mg/L), white blood cell count (6.7 Gpt/L), blood gas analysis (ph=7.43, p(CO_2_)=33 mmHg, [natrium]=133 mmol/L, anion gap 4.8 mmol/L, other parameters in normal range); beginning of a calculated antimicrobial therapy with meropenem and vancomycin intravenously
2	stool samples on pathogenic bacteria and viruses, cultural detection of a Gram-negative rod in the blood culture bottles (time to positivity 5h 17 min)
4	availability of a preliminary identification of the Gram-negative rod as *Pantoea* sp. related species (Vitek2 GN card, 94%, no identification using Vitek MS) and preliminary susceptibility testing results by MIC-test strips for meropenem (MIC=0.032 mg/L) and ceftazidime (MIC=0.5 mg/L) both tested susceptible
6	vancomycin intravenously was terminated according to susceptibility testing, stool samples on pathogenic bacteria (including *Clostridioides difficile*) and viruses were reported “negative”, last day of elevated body temperature (38.0°C)
10	complete regression of fever
12	further stool samples on pathogenic bacteria (including *Clostridioides difficile*) and viruses were reported “negative**”**
14	follow-up blood cultures were reported “negative**”**, meropenem intravenously was terminated
17	discharge from hospital

### 4.3 Clinical manifestations in humans

*Chimaeribacter arupi*, as a member of the order Enterobactereales and the family Yersiniaceae, appears to have its primary habitat in the intestine of humans. It has not yet been proven whether it is introduced here through food from a zoonotic host or if it occurs exclusively in humans. Two samples in which *Chimaeribacter* (“*Nissabacter*”) species were detected were of aerial origin (2 of 12) in the broadest sense, i.e. for one sample the bacterial isolate is known to have originated from a *Drosophila* fly that acted as a mechanical vector and possibly had prior contact with feces (Gaultier et al., 2019). The other *Chimaeribacter* (“*Nissabacter*”) isolates originate from wounds (4 of 12) and blood cultures (5 of 12, including our isolate). A single *Chimaeribacter arupi* isolate was obtained from respiratory material of a patient with chronic sinusitis and pneumonia (1 of 12) (Mlaga et al., 2017; Rossi and Fisher, 2020). This detection pattern in clinical samples suggests contamination of existing wounds with feces and bloodstream infections resulting from invasive migration through the intestinal epithelium.

### 4.4 Genome quality in comparison to other *C. arupi* genomes

**Figure 2:**
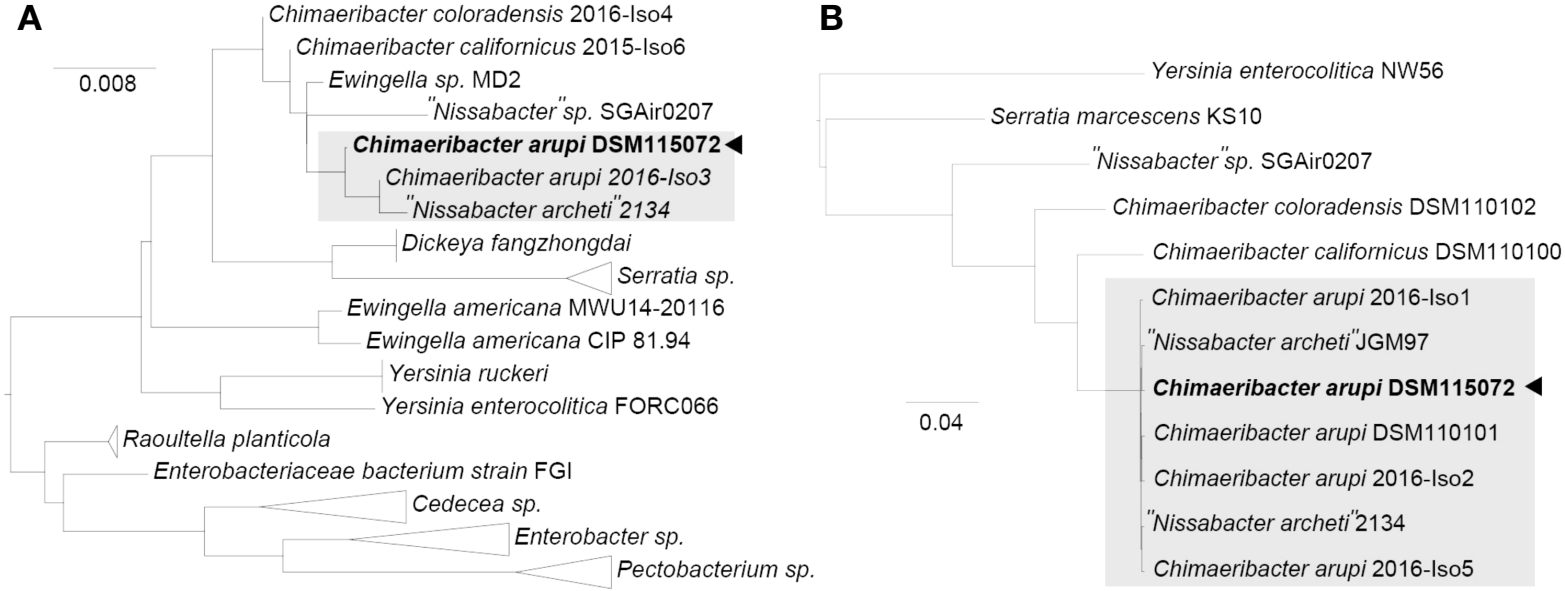
Midpoint-rooted maximum-likelihood tree from **(A)** ClustalW alignments of BLASTn results for *C*. *arupi* DSM115072 full length 16S rDNA against NCBI 16S ribosomal RNA sequence database and from **(B)** Mafft alignments of 284 core-genes (*Y. enterocolitica* and *S. marcescens* as outgroups), as determined by Panaroo.

The consensus assembly using “Trycycler” from 20 independent long read (LR) assemblies (4x “Flye” LR assembly, 4x “miniasm/minipolish” LR assembly, 4x “necat” LR assembly, 4x “nextdenovo/nextpolish” LR assembly, and 4x “raven” LR assembly) combined with long read (“Medaka”) & short read (“Polypolish”) polishing resulted in four contigs. The longest contig corresponds to the circular closed chromosome with a length of 4,371,535 bp. The second and third largest contigs were two circular plasmids of a length of 352,560 bp (p353_DSM115072) and 250,769 bp (p251_DSM115072), respectively. The fourth and smallest contig corresponds to a linear extrachromosomal element 37,771 bp in length (linear extrachromosomal element DSM115072). Taxcheck using “fastANI” identified our isolate as closely related to “*Nissabacter archeti*” strain 2134 (NCBI RefSeq assembly: GCA_900130115.1; BioProject: PRJEB18266) with 99.346% sequence identity, *C. arupi* strain 2016-Iso3 (NCBI RefSeq assembly: GCA_002858805.1; BioProject: PRJNA422155) with 99.336% sequence identity, respectively. This taxonomic classification of our clinical isolate is further confirmed and graphically visualized by a phylogenetic tree based on ClustalW alignments of BLASTn results for the full-length sequence of the 16S rRNA gene against NCBI 16S ribosomal RNA sequence database (**Figure 2A**). In a second phylogenetic tree we show Mafft alignments of 284 core-genes of all *Chimaeribacter* and “*Nissabacter*” genome sequences, which are currently accessible including *Y. enterocolitica* and *S. marcescens* as outgroups (**Figure 2B**). In addition to confirming the phylogenetic classification of our isolate, these dendrograms respectively analyses reconfirm that *Chimaeribacter arupi* and “*Nissabacter archeti*” are heterotypic synonyms for one and the same microbiological species.

The genome of *C. arupi* DSM115072 is currently the only complete and closed genome with the highest sequence quality, which is also supported by quality parameters such as contig N50 of 4.37 Mb, contig L50 of 1 and a genome coverage of 57x (see **Table 4**).

**Table 4:** Overview of all currently accessible *Chimaeribacter arupi* and “*Nissabacter archeti*” genomes including their sequence quality parameters.

Name	Assembly level	Genome size	Genome coverage	No. of contigs	Contig N50	Contig L50
*Chimaeribacter arupi* 2016-Iso2	contig	5.0 Mb	23x	101	136.6 kb	14
*Chimaeribacter arupi* 2016-Iso5	contig	4.8 Mb	24x	118	144.3 kb	11
*Chimaeribacter arupi* DSM110101	contig	4.9 Mb	19x	121	98.5 kb	16
*Chimaeribacter arupi* 2016-Iso1	contig	4.7 Mb	30x	80	180.8 kb	8
“*Nissabacter archeti*” JGM97	scaffold	5.0 Mb	185x	30	287.5 kb	7
“*Nissabacter archeti*” 2134	contig	5.2 Mb	39x	50	262.7 kb	7
*Chimaeribacter arupi* DSM115072	complete	5.0 Mb	57x	4	4.37 Mb	1

### 4.5 Description of genome content (incl. Comparative analyses)

#### 4.5.1 Genome analysis

**Figure 3:**
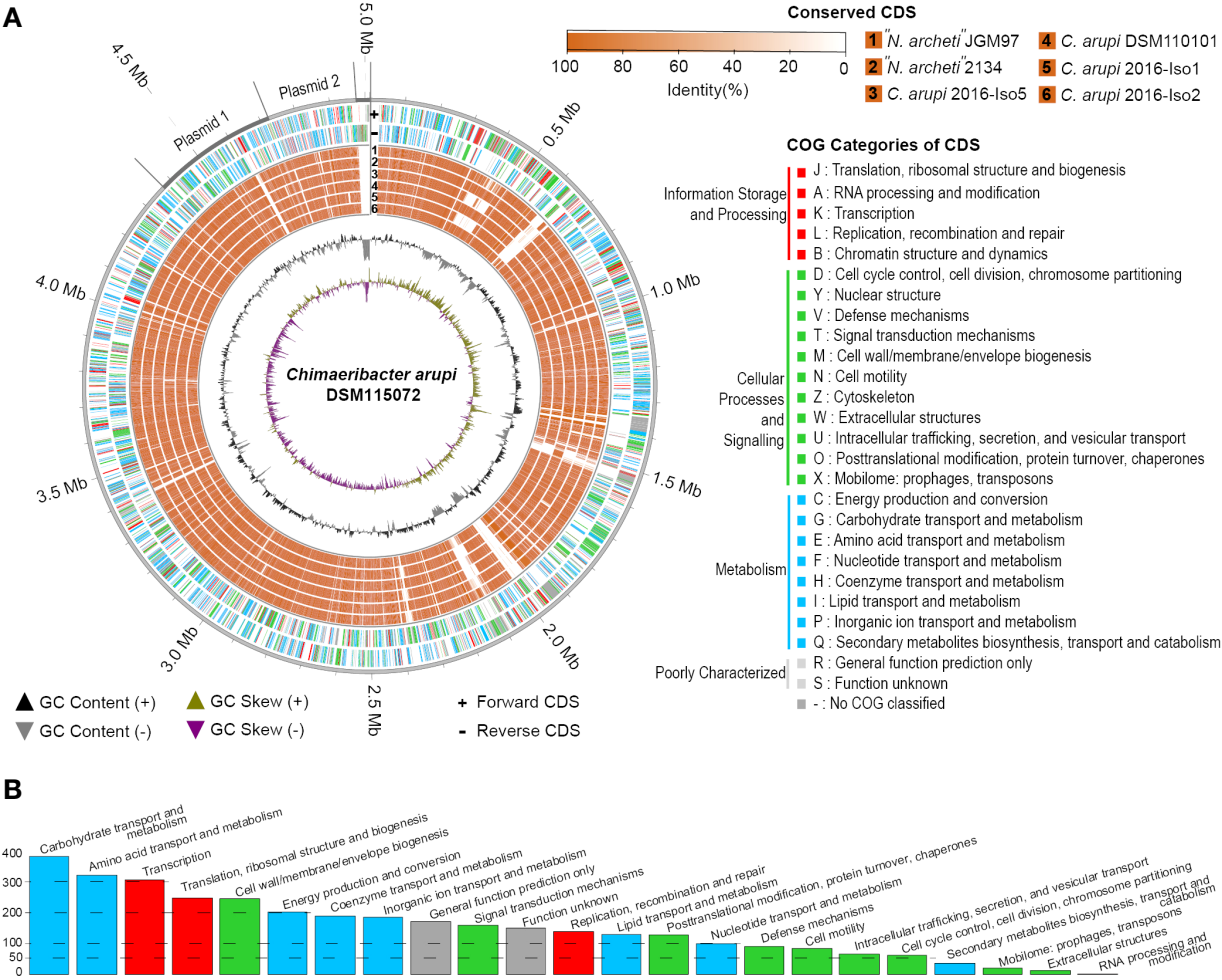
**(A)** Genome circos plot of conserved CDS between *C. arupi/”N. archeti*” genomes vs *C*. *arupi* DSM115072 on Chromosome, Plasmid1 (p353_DSM115072), Plasmid 2 (p251_DSM115072), and linear extrachromosomal element DSM115072. Reciprocal BLAST Best Hits analysis (RBH) was performed to identify conserved CDS. Plot generation, COG identification and RBH analysis was performed using MGCplotter. **(B)** Barplot of COG categories present in *C*. *arupi* DSM115072.

The G+C content of the chromosome was GC % = 58.2%. As indicated in **Figure 3** the GC skew of the *C. arupi* DSM115072 genome was predominantly positive in the region 0 bp – 2.28 Mbp but predominantly negative in the region 2.28 Mbp – 4.37 Mbp indicating a switch of the leading strand (**Figure 3A**).

**Figure 4:**
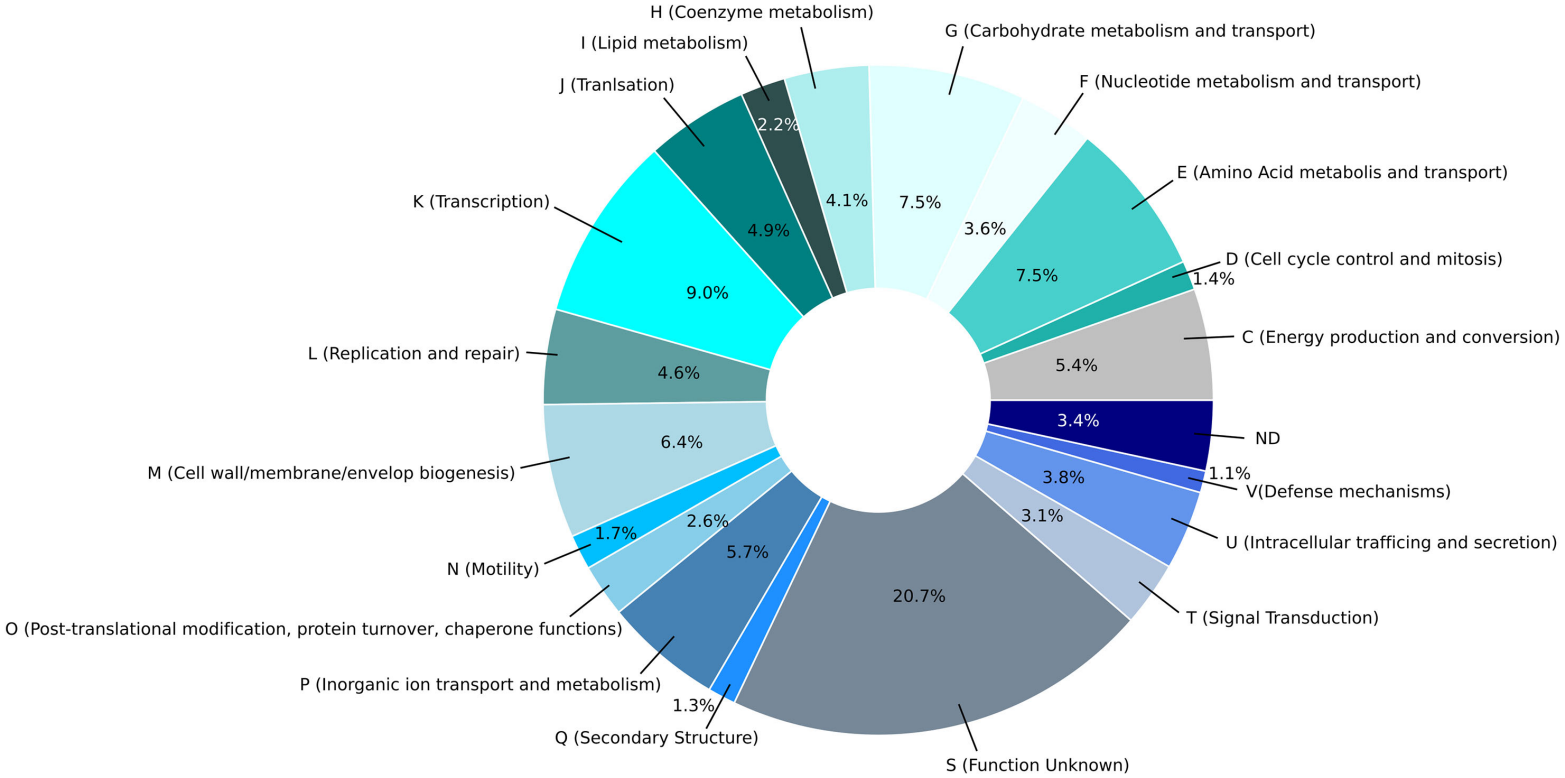
Pie chart of the percentage distribution of the COG categories to which individual CDSs in the genome of *C. arupi* DSM115072 was assigned. The 20 most abundant categories level identified by EggNOG 5.0 are represented by a specific color.

Application of the NCBI-annotation pipeline resulted in 4,574 genes. Of this total number of genes, 4,332 are proteincoding genes, 127 are pseudo-genes, and 115 are RNA-coding genes. Out of these RNA-coding genes, 82 encode tRNAs, 11 encode ncRNAs, 8 encode 5S rRNAs, 7 encode 16S rRNAs, and 7 encode 23S rRNAs. A functional prediction of protein function was performed using EggNOG 5.0. The clusters of orthologous genes (COG) classification of the genetic features of each protein-coding gene are indicated in **Figure 3A** and a histogram in **Figure 3B** shows the absolute number of CDS in specific COG categories. Additionally, the percentage distribution of the COG categories to which individual CDSs in the genome of *C. arupi* DSM115072 could be assigned is depicted in **Figure 4**. Apart from not being able to assign 20.7% of CDS to any COG category, the largest functional categories are transcription (9.0%), carbohydrate metabolism and transport (7.5%), amino acid metabolism and transport (7.5%), cell wall/membrane/envelope (6.4%), inorganic ion transport and metabolism (5.7%), and energy production and conversion (5.4%).

The three extrachromosomal elements of *C. arupi* DSM115072 are also present in the other six currently accessible *C. arupi*/”*N. archeti*” genomes. The largest extrachromosomal element is a circular plasmid of a length of 352,560 bp (p353_DSM115072). It is difficult to give the plasmid a functional title, according to the genes or proteins encoded by the coding sequences on it. The term “stress response and habitat establishment” could be used to group together the plasmid functions. The plasmid contains several genes, including those for tellurium resistance, macrolide resistance (such as the macrolide-specific efflux proteins MacA/B), and Phenazine biosynthesis (PhzF). Phenazines are nitrogen-containing aromatic compounds that have antibiotic properties. Many bacteria produce and release these compounds into their environment to protect their habitat. Two genes encode for a manganese catalase, which may protect the bacterial cell from oxidative stress. The active site of manganese catalase contains two manganese ions that are transported by a manganese ABC transporter. This transporter consists of four proteins encoded by *sitD*, *sitC*, *sitB*, and *sitA* genes located on the plasmid. Additionally, there are two other proteins, YedW and YedV, that form a putative two-component system. YedW is a response regulator, while YedV is a histidine kinase that plays a role in regulating the response to H_2_O_2_ (Urano et al., 2015). Moreover, the plasmid contains the genes *lpfA*/*B*/*C*/*D*, which encode long polar fimbria and facilitate attachment to epithelial cells, enabling colonization of the intestine. This mechanism has also been observed in other bacteria such as *Escherichia coli* O157:H7 and *Salmonella enterica* serovar Typhimurium (Jordan et al., 2004). Additionally, the plasmid encodes various ABC transporters, including those responsible for transporting gamma-aminobutyric acid.

The second largest circular plasmid of a length of 250,769 bp (p251_DSM115072) encodes predominantly enzymes or proteins that play a role in amino acid and carbohydrate metabolism.

The smallest extrachromosomal element is 37,771 bp in length (linear extrachromosomal element DSM115072). Apart from hypothetic proteins, it was only possible to annotate the genes for the plasmid partitioning protein ParA, the chaperone protein DnaK, error-prone repair protein UmuD, and a RelE-like translational repressor toxin. Therefore, the function of this linear extrachromosomal element remains largely unclear.

The genome of *C. arupi* DSM115072 contains four regions that encode phage proteins. One region is located on the chromosome at position 1,001,607..1,025,368 bp and spans 23.7 Kb, consisting of 30 CDS encoding phage proteins (**Supplementary Table 2**). Another region is found on plasmid p353_DSM_115072, spanning 10.9 Kb at position 223,455..234,443 bp, comprising 10 CDS encoding phage proteins (**Supplementary Table 5**). Both of these phages are incomplete. Additionally, there is a region on the chromosome at position 1,500,941..1,545,284 bp that spans 44.3 Kb, consisting of 56 CDS (**Supplementary Table 3**), which PHASTER has identified as an intact phage (Rossi and Fisher, 2020). Another larger region at 2,081,008..2,132,473 bp, spanning 51.4 Kb, is also present on the chromosome. PHASTER has labeled this region as encoding a “questionable” phage, with only 36 phage protein-encoding CDSs identified. However, using the NCBI Annotations Pipeline, the number of annotated CDSs can be increased to 53, which raises the possibility that it is also an intact phage (**Supplementary Table 4**).

#### 4.5.2 Antimicrobial resistance genes

Considering the phenotypic resistance for ampicillin, we expected at least one beta-lactamase. Although ResFinder 4.1 was unable to identify a resistance gene in the *C. arup*i DSM115072 genome, ResFinderFG 2.0 was able to detect a beta-lactam resistance determinant at position 545,630..547,063 (locus_tag=“P0E69_02605”). The coding sequence located there encodes a protein with the predicted function serine-type D-Ala-D-Ala carboxypeptidase (GO:00041859) that is related to the penicillin sensitive murein endopeptidases DacB in *Escherichia coli*. Using the NCBI annotation pipeline, a class A beta-lactamase gene (*bla*, GO:0008800) at position 234,442..235,329 (locus_tag=“P0E69_22805”) could be located. Additionally, the beta-lactamase regulator genes, *ampE* at position 3,591,824.3,592,678 (locus_tag=“P0E69_16525”), and *ampD* at position 3,592,723..3,593,274 (locus_tag=“P0E69_16530”) were located in the genome (Tuomanen et al., 1991). Of note, there is a metallo-beta-lactamase (frameshifted) pseudogene at position 110,373..111,178 (locus_tag=“P0E69_20525”) on plasmid p353_DSM_115072.

Finally, ResFinderFG 2.0 located a D-alanine–D-alanine ligase gene, *ddlA*, at position 3,022,699..3,021,602 (locus_tag=“P0E69_13810”) that potentially may play a role in conferring resistance to D-cycloserine, which was not tested phenotypically.

In addition, various MDR efflux pumps are located in the genome, including the macrolide-specific efflux protein MacA and macrolide export ATP-binding/permease protein MacB, which have already been mentioned under extrachromosomal elements.

#### 4.5.3 Virulence factors

One key factor that determines the virulence of a pathogen is its ability to produce capsular polysaccharides or form a protective capsule that shields against phagocytosis and environmental stress. In the genome of *C. arupi* DSM115072, a complete gene locus for capsular polysaccharide synthesis (*cps* locus) is present at position 2,963,965..2,977,011, consisting of seven characteristic genes: *kpsM*, *kpsT*, *kpsS*, *kpsC*, *kpsD*, *kpsF*, and *kpsE*. Downstream of *cps*, a two-component system regulates the expression of *cps* and additionally of the cell division gene *ftsZ* (P0E69_03395). This two-component system is composed of the histidine kinase RcsC (P0E69_06175) and the DNA-binding capsular synthesis response regulator RcsB (P0E69_06180). Additionally, there is the regulatory protein RcsA (P0E69_07215) that binds with RcsB to the RcsAB box, controlling the expression of genes that play a role in capsule synthesis. (Wehland and Bernhard, 2000). A fourth component is RcsD (P0E69_06185), a phosphotransferase that transfers a phosphoryl group from the sensor kinase RcsC (P0E69_06175) to the response regulator RcsB (P0E69_06180) (Takeda et al., 2001).

Additionally, *C. arupi* DSM115072 possesses a comprehensive set of enzymes required for the synthesis of lipopolysaccharide (LPS; **Table 5**). This includes the zinc metalloprotease FtsH, which plays a crucial role in regulating the production rate of lipid A by degrading LpxC and WaaA, as well as the FtsH-regulating proteases HflK, YccA, and the FtsH-inhibitory lysogeny factor CIII (Emiola et al., 2015). LPS serves as a vital component of the outer membrane in Gram-negative bacteria, contributing significantly to the structural integrity of the bacteria and providing protection against certain types of stressors. Toll-like receptor 4 (TLR4) recognizes LPS, ultimately leading to an increase in TNF-α, which can trigger a general activation of the immune system and potentially result in the development of sepsis (Richard et al., 2020).

**Table 5:** Enzymes involved in LPS synthesis of *C. arupi* DSM115072.

Locus tag	Gene	Position	Predicted function
P0E69_04330	*lpxA*	934570..935358	acyl-ACP–UDP-N-acetylglucosamine O-acyltransferase
P0E69_04335	*lpxB*	935362..936510	lipid-A-disaccharide synthase
P0E69_16595	*lpxC*	3605154..3606071	UDP-3-O-acyl-N-acetylglucosamine deacetylase
P0E69_04320	*lpxD*	932932..933954	UDP-3-O-(3-hydroxymyristoyl)glucosamine N-acyltransferase
P0E69_14790	*lpxH*	3219078..3219800	UDP-2,3-diacylglucosamine diphosphatase
P0E69_13000	*lpxK*	2827698.2828690	tetraacyldisaccharide 4’-kinase
P0E69_12435	*lpxL*	2717725..2718645	LpxL/LpxP family Kdo(2)-lipid IV(A) lauroyl/palmitoleoyl acyltransferasee”
P0E69_09230	*lpxM*	2054996..2055976	lauroyl-Kdo(2)-lipid IV(A) myristoyltransferase
P0E69_18015	*lpxP*	3914352..3915272	kdo(2)-lipid IV(A) palmitoleoyltransferase
P0E69_19540	*waaA*	4260798..4262075	lipid IV(A) 3-deoxy-D-manno-octulosonic acid transferase
P0E69_02625	*ftsH*	549058..550989	ATP-dependent zinc metalloprotease FtsH, degradation of LpxC and WaaA
P0E69_02385	*hflK*	502237..503484	FtsH protease activity modulator HflK
P0E69_06705		1507197..1507328	protease FtsH-inhibitory lysogeny factor CIII
P0E69_12750	*yccA*	2770866..2771522	FtsH protease modulator YccA
P0E69_13005	*msbA*	2828687..2830435	lipid A ABC transporter ATP-binding protein/permease MsbA

Gram-negative bacteria have evolved various types of protein secretion systems, including the type 6 secretion system (T6SS). The T6SS consists of several components, such as a membrane complex that spans both membranes and is assembled from TssJ, TssL, and TssM. Additionally, there is a baseplate complex composed of TssA, TssE, TssF, TssG, TssK, and valine-glycine repeat protein G (VgrG or TssI). Furthermore, there is a contractile sheath made up of TssB and TssC, along with a spike complex that includes a trimer of VgrG. This trimer acts as a cell puncturing device, facilitating the injection of several effector proteins into the target cell (Gallique et al., 2017; Cherrak et al., 2019; Lin et al., 2019). Among these effector proteins is the hemolysis co-regulated protein Hcp that has the ability to lyse erythrocytes. The energy required for protein transport across cellular barriers is provided by two potential ATPases, TssM (IcmF) and ClpV (TssH) (Bleumink-Pluym et al., 2013; Basler, 2015). In the genome of *C. arupi* DSM115072 we found T6SS encoding proteins at three different loci. At position 1,414,652..1,443,896 we found a gene cluster encoding 23 T6SS-associated coding sequences including *tssB*/*impB*/*vipA* (P0E69_06295), *tssC*/*impC*/*vipB* (P0E69_06300), *tssK*/*impJ*/*vasE* (P0E69_06305), *tssL*/*impK*/*vasF* (P0E69_06310), *hcp* (P0E69_06320), *tssH* (P0E69_06325), *vgrG* (P0E69_06330), *tssM*/*icmF*/*vasK* (P0E69_06365), *tssA*/*impA* (P0E69_06370), *tssF*/*impG*/*vasA* (P0E69_06375), *tssG*/*impH*/*vasB* (P0E69_06380), *tssJ* (P0E69_06385), *tssE* (P0E69_06390), and *tssA*/*vasL* (P0E69_06395) and several CDS encoding hypothetical proteins. These 23 CDS make up a complete T6SS. A second gene cluster with 28 T6SS-associated coding sequences was found at position 2357858..2381595. The coding sequences of this gene locus include *tssJ*/*vasD* (P0E69_10675), *tssG*/*impH*/*vasB* (P0E69_10680), *tssF*/*impG*/*vasA* (P0E69_10685), *tssM*/*icmF*/*vasK* (P0E69_10690), *tssM*/*icmF*/*vasK* (P0E69_10695), PAAR domain-containing protein (P0E69_10700), *vgrG* (P0E69_10735), *hcp* (P0E69_10750), *ompA* family protein (P0E69_10755), *tssL*/*impK*/*vasF* (P0E69_1076), *tssK*/*impJ*/*vasE* (P0E69_10765), *tssC*/*impC*/*vipB* (P0E69_10770), and *tssB*/ImpB/VipA (P0E69_10775). For a fully functional T6SS apparatus, the following genes were absent: *tssH*, *tssA*, and *tssE*. However, we observed the presence of a PAAR domain-containing protein, an additional effector protein, and a CDS encoding an OmpA family protein, which is not typically associated with a T6SS. It has been demonstrated that the accessory protein TagH in *Pseudomonas aeruginosa* is involved in the posttranslational regulation of the assembly of the T6SS. This protein is phosphorylated, thus regulated in its activity, by a serine-threonine kinase called PpkA. Additionally, it is dephosphorylated by a phosphatase known as PppA (Mougous et al., 2007). At position 1,850,195..1,854,966 in the *C. arupi* DSM115072 genome we found a gene cluster including *ppkA* (P0E69_08390), *pppA* (P0E69_08400) and a gene for the T6SS forkhead associated domain protein TagH/ImpI/VasC (P0E69_08405). And last, we found a single *hcp* gene (P0E69_10400) at 2,295,263..2,295,745.

Furthermore, the genome of *C. arupi* DSM115072 contains genes encoding a predicted hemolysin (P0E69_02495), a predicted type III hemolysin (P0E69_03880), and a predicted hemolysin activator protein (P0E69_02770; **Table 6**). The gene products of these CDS as well as the T6SS secreted Hcp could potentially cause the observed weak hemolysis after prolonged culture.

**Table 6:** Hemolysin genes in the genome of *C. arupi* DSM115072.

Locus tag	Gene	Position	Predicted function
P0E69_02495		523456..524799	hemolysin family protein
P0E69_02770		579462..580712	ShlB/FhaC/HecB family hemolysin secretion/activation protein
P0E69_03880		832440..833095	hemolysin III family protein (pseudogene)

Many Gram-negative bacteria possess various types of hair-like pili, also known as fimbriae, which typically facilitate adhesion to different surfaces or mediate interactions with other bacteria or eukaryotic cells of the host organism. Among these, pili assembled through the chaperone/usher (CU) pathway are quite common. This pathway relies on a periplasmic chaperone and an “usher,” an integral outer membrane protein that serves as a versatile assembly machinery with secretory functions. The fimbrial subunits are directed to the Sec-secretory pathway by an *N*-terminal signal sequence and transported to the periplasmic space. Once there, the signal sequence is cleaved through proteolysis. The periplasmic oxidoreductase DsbA (in *C. arupi* DSM115072: P0E69_19725) facilitates the closure of disulfide bridge-bonds in the subunits. Following this, the periplasmic chaperone FimC folds the fimbrial subunits into Ig-like domains. Misfolded and aggregated subunits are eliminated by the protease DegP (in *C. arupi* DSM115072: P0E69_16310). The individual subunits are then transported outside through the usher and connected in parallel to form a helical structure of Ig-like domains (Werneburg and Thanassi, 2018). Pili of the chaperone/usher pathway are divided into the clades α, β, γ, κ, π, and σ according to their phylogenetic relationship (Nuccio and Bäumler, 2007). In the genome of *C. arupi* DSM115072, we identified six pili belonging to the γ-clade, as well as one each from the α- and σ-clades. The prototype of γ_1_-pili, also called type 1 pili according to the old nomenclature, was found in *E. coli* and the operon consists of the genes *fimBEAICDFGH*. The spiral-shaped pilin rod proximal to the cell surface is formed from FimA, the major pilin subunit also called fibrillin. FimF and FimG are adapter subunits found in the distal tip fibrillum, while FimH serves as the adhesin associated with the tip. FimC acts as the pilus chaperone, and FimD functions as the usher protein. FimI is likely the subunit responsible for terminating the rod. FimB and FimE are regulatory proteins involved in the phase variation of type 1 pilus expression. It should be noted that not all pili operons are as complete as this one. The six type 1/γ_1_-pili operons of *C. arupi* DSM115072 are listed in **Table 7**. In addition to predicted function, orthologous genes (obtained using Delta-BLAST) in other microbial species are also listed there. At position 199,097..203,992 we find a type 1/γ_1_-pili operon consisting of only four genes, which, in addition to the genes for fimbrillin, chaperone and usher, also contains a *fimF* related gene, which possibly, not only has an adapter function but also an adhesin function. At positions 572,887..577,873 and 1,976,516..1,981,284, we discovered two additional type 1-pili operons consisting of four genes. However, the function of the gene product of the fourth gene remains unclear based on BLAST analyses. It is possible that this protein is located at the tip of the pili. At genome position 1,745,203..1,750,119, there is a type 1-pili operon consisting of five genes. Within this operon, there is a putative protein that is potentially localized at the tip of the pilus. However, based on the gene sequence, it is not clearly assignable to other orthologous genes in other microbial species. Additionally, there is a second protein predicted to function as an outer membrane usher protein. Type 1 pili of this type are rigid, mediate mannose-sensitive hemagglutination and binding to various surfaces and tissues via a mannose-sensitive manner (Werneburg and Thanassi, 2018). A six-gene type 1 pilus operon is found at position 2,987,004..2,994,492. In addition to the genes for fimbrillin, chaperone and usher, there were three genes with gene products that show a higher similarity to the fimbrial protein of the long polar (LP) fimbria of *Salmonella enterica* serovar Typhimurium. Thus, this operon also has more structural similarity to the *lpfABCC′DE*operon. The LP fimbria play a role in interaction with eukaryotic cells in particular mediating adhesion to the M cells of Peyer’s patches, which may also be the case in *C. arupi* (Torres et al., 2002; Werneburg and Thanassi, 2018). A principally complete *yadCKLM-htrE-yadVN* homologous operon is located at position 3,112,790..3,118,678. However, the function of these pili is still unclear.

**Table 7:** Overview of the pili operons of the γ-clade in the genome of *C. arupi* DSM115072.

Locus tag	Gene	Position	Predicted function
P0E69_00840	*fimA*/*lpfA*	199097..199654	type 1 major fimbrial protein, fimbrillin (mannose-sensitive hemagglutiantion, gamma-fimbriae)
P0E69_00845	*gimC/lpfB//papD*/*ecpD*	199713..200423	molecular chaperone
P0E69_00850	*fimD*	200467..202971	fimbrial biogenesis outer membrane usher protein, type 1 fimbriae anchoring protein FimD
P0E69_00855	*fimF*	202985..203992	type 1 fimbrial protein (tip, adapter subunit )?
P0E69_02740	*lpfA/fimA*	572887..573462	type 1 major fimbrial protein, fimbrillin (mannose-sensitive hemagglutiantion, gamma-fimbriae)
P0E69_02745	*fimC*	573512..574228	fimbria/pilus periplasmic chaperone
P0E69_02750	*fimD*	574264..576861	fimbrial biogenesis outer membrane usher protein
P0E69_02755	?	576893..577873	type 1 fimbrial protein
P0E69_07855	*fimA*/*sfaA*	1745203..1745748	type 1 major fimbrial protein, fimbrillin
P0E69_07860	?	1745749..1747581	fimbrial biogenesis outer membrane usher protein (*Rahnella aquatilis*)
P0E69_07865	*fimD*	1747626..1748303	fimbria/pilus outer membrane usher protein
P0E69_07870	*fimC*	1748348..1749082	fimbria/pilus periplasmic chaperone
P0E69_07875	?	1749079..1750119	type 1 fimbrial protein
P0E69_08860	?	1976516..1977535	type 1 fimbrial protein
P0E69_08865	*fimD*	1977556..1979949	fimbrial biogenesis outer membrane usher protein
P0E69_08870	*fimC*	1979961..1980656	molecular chaperone
P0E69_08875	*fimA*	1980715..1981284	type 1 major fimbrial protein, fimbrillin
P0E69_13645	*fimA*/*lpfA*	2987004..2987528	type 1 major fimbrial protein, fimbrillin
P0E69_13650	*flimC*	2987837..2988523	molecular chaperone
P0E69_13655	*fimD*	2988590..2991160	fimbrial biogenesis outer membrane usher protein
P0E69_13660	*lpfD*, *stgD*	2991229..2992260	fimbrial protein, minor fimbrial subunit, adhesion domain
P0E69_13665	*lpfD*	2992332..2993402	fimbrial protein
P0E69_13670	*lpfD*	2993452..2994492	fimbrial protein
P0E69_14260	*yadC*/*stkG*/*fimA*	3111710..3112777	Type 1 fimbrial major protein
P0E69_14265	*yadK*/*staF*	3112790..3113374	fimbrial-like protein
P0E69_14270	*yadL*	3113405..3113965	fimbrial protein
P0E69_14275	*yadM*/*fimA*/*sfmA*	3113982..3114560	fimbrial protein
P0E69_14280	*htrE*	3114651..3117197	outer membrane usher protein
P0E69_14285	*ecpD*	3117288..3118022	fimbrial chaperone EcpD
P0E69_14290	*yadN*	3118091..3118678	fimbrial-like protein YadN part of the *yadCKLM-htrE-yadVN* fimbrial operon.

In addition to the various γ_1_-pili, there was also a TcfC-like (CS1 type) pilus from the α-clade in the *C. arupi* DSM115072 genome (**Table 8**). TcfC-like pili are found in pathogens that are highly adapted to humans, such as *S. enterica* serotype Typhi and *S. enterica* serotype Paratyphi A. Unfortunately, these chromosomally encoded pili have not yet been fully functionally characterized, but they appear to play a role in selective binding to human tissues (Nuccio and Bäumler, 2007). Consequently, it is likely that the human intestine should be habitat of *C. arupi*. This was confirmed by a BLAST search using the 16S rRNA gene sequence of *C. arupi* against the human gut metagenome 16S ribosomal RNA database. This BLAST search resulted in 100 hits with a query coverage ranging from 35% to 90% and a sequence identity of 77.93% to 95.47%.

**Table 8:** CDSs of the α-pilus operon of *C. arupi* DSM115072.

Locus tag	Gene	Position	Predicted function
P0E69_15555	*cfaE*/*cblD*	3384465..3385340	CfaE/CblD family pilus tip adhesin, Alpha-fimbriae tip adhesin
P0E69_15560		3385456..3386187	fimbria/pilus periplasmic chaperone, Alpha-fimbriae chaperone protein
P0E69_15565		3386293..3386811	CS1 type fimbrial major subunit, Alpha-fimbriae major subunit
P0E69_15570	*tcfC*	3386896..3389589	TcfC E-set like domain-containing protein, Alpha-fimbriae usher protein
P0E69_15575	*cfaE*/*cblD*	3389586..3390731	CfaE/CblD family pilus tip adhesin, Alpha-fimbriae tip adhesin

Moreover, the genome of *C. arupi* DSM115072 also includes an operon that encodes for the proteins of a σ-pilus (**Table 9**). Pili from this group are considered archaeal and are found across various phylogenetic lineages. However, their functions remain largely understudied. For instance, σ-pili are involved in biofilm formation and the formation of spore coats.

**Table 9:** CDSs of the σ-pilus operon of *C. arupi* DSM115072.

Locus tag	Gene	Position	Predicted function
P0E69_11830		2597158..2597688	Sigma-fimbriae tip adhesin, spore coat U domain-containing protein
P0E69_11835		2597700..2598251	Sigma-fimbriae uncharacterized paralogous subunit, spore coat U domain-containing protein
P0E69_11840		2598268..2598825	Sigma-fimbriae uncharacterized subunit, spore coat U domain-containing protein
P0E69_11845		2598855..2599619	Sigma-fimbriae chaperone protein
P0E69_11850		2599582..2602548	Sigma-fimbriae usher protein
P0E69_11855		2602584..2603534	Sigma-fimbriae tip adhesin, spore coat protein U domain-containing protein

In addition to the eight different CU pili, the genome of *C. arupi* DSM115072 contains all necessary CDS for a complete Type IV “pilus nanomachine” distributed over the whole genome. Type IV pili are present in many Gram-negative bacteria that are known to cause human infections and are considered to be ubiquitous. They have been extensively studied, particularly in *Pseudomonas aeruginosa*. The predicted assembly process of type IV pili in *C. arupi* DSM115072 can be simplified as follows (**Table 10**): The type IV pili are composed of a polymerized major pilin, which is encoded by the *pilA* gene (P0E69_16540), along with related proteins (P0E69_04110, P0E69_04115, P0E69_04125, P0E69_22930, P0E69_23080), and minor pilins (PilW, P0E69_04985). The prepilin peptidase PilD (P0E69_01340), located in the membrane, processes the pre-pilins into their mature form, enabling polymerization. Pilin polymerization is actively facilitated by an assembly-ATPase called PilB (P0E69_16545) and an inner membrane platform protein called PilC (P0E69_16550) (Ellison et al., 2022). In Gram-negative bacteria, the pilus needs to traverse the outer membrane. Therefore, the presence of a secretion protein localized in the outer membrane is necessary. The inner membrane accessory proteins PilN (P0E69_01140), PilM (P0E69_01135), and PilO (P0E69_01145), along with the outer membrane secretin PilQ (P0E69_01155), form a channel through which the pilus grows. The periplasmic domains of the inner membrane accessory proteins PilN and PilO interact with the secretin channel through PilP (P0E69_01150), creating a periplasmic conduit for pilus growth (Melville and Craig, 2013). For pilus retraction and the twitching motility facilitated by type IV pili, a retraction ATPase called PilT (P0E69_03645) is required (Ellison et al., 2022).

**Table 10:** CDSs encoding Type IV pilus proteins on *C. arupi* DSM115072.

Locus tag	Gene	Position	Predicted function
P0E69_01135	*pilM*	272261..273091	pilus assembly protein PilM
P0E69_01140	*pilN*	273088..273768	PilN domain-containing protein
P0E69_01145	*pilO*	273740..274174	pilus assembly protein PilO
P0E69_01150	*pilP*	274186..274530	Type IV pilus inner membrane component PilP
P0E69_01155	*pilQ*	274721..275950	type IV pilus biogenesis protein PilQ, Required for type IV pilus biogenesis and competence. Could function as a pore for exit of the pilus but also as a channel for entry of heme and antimicrobial agents and uptake of transforming DNA (By similarity)
P0E69_01160	*aroK*	276337..276858	shikimate kinase AroK
P0E69_01165	*aroB*	276928..278019	3-dehydroquinate synthase
P0E69_01340	*pilD*	308183..308977	leader peptidase (Prepilin peptidase), A24 family peptidase
P0E69_03645	*pilT*	779166..780167	type IV pilus twitching motility protein PilT
P0E69_04110		885845..886306	prepilin peptidase dependent protein A precursor; prepilin-type cleavage/methylation domain-containing protein
P0E69_04115		886297..886884	prepilin peptidase dependent protein B precursor; prepilin peptidase-dependent protein
P0E69_04125		887318..887650	prepilin-type N-terminal cleavage/methylation domain-containing protein
P0E69_04985	*pilW*	1065003..1065776	type IV pilus biogenesis/stability protein PilW
P0E69_16540	*pilA*/*ppdD*	3594521..3594958	type IV pilin PilA, prepilin peptidase-dependent pilin
P0E69_16545	*pilB*/*gspE*	3594955..3596388	type IV fimbrial assembly, ATPase PilB, type II secretion system protein GspE
P0E69_16550	*pilC*/*hofC*	3596381..3597586	type IV fimbrial assembly protein PilC, protein transport protein HofC
P0E69_22930		7932..8474	type IV pilus major pilin
P0E69_22935		8487..9044	type IV pilus major pilin
P0E69_23080		31851..32372	type IV pilus major pilin

Type IV pili play a crucial role in the attachment of bacteria to host cells, abiotic surfaces, and other bacteria. This is important for the formation of biofilms and colonization in the intestines during the onset of infection. Type IV pili are also involved in bacterial conjugation, which is the transfer of genetic material, such as plasmids, between bacteria. Additionally, they are involved in the uptake of DNA during natural transformation, allowing bacteria to acquire antibiotic resistance and virulence genes. Twitching motility is another important capability facilitated by type IV pili, enabling bacteria to move across surfaces by extending, binding, and retracting the pili. Furthermore, type IV pili are involved in the secretion of proteins, including virulence factors, across the bacterial membrane, similar to flagella which have an integrated type III secretion system (Craig and Li, 2008; Melville and Craig, 2013; Ellison et al., 2022). It is reasonable to assume that the type IV pili nanomachine of *C. arupi* fulfills similar functions.

#### 4.5.4 Further virulence associated factors

The bacterium *C. arupi* DSM115072 has a full set of genes that encode enzymes involved in the biogenesis of Gram-negative bacterial cell walls or the biosynthesis of peptidoglycan (**Table 11**). These genes are not arranged in a cluster, but are distributed throughout the entire genome.

**Table 11:** Enzymes involved in Gram-negative-bacterium-type cell wall biogenesis and peptidoglycan biosynthesis.

Locus tag	Gene	Position	Predicted function
P0E69_17720	*murA*	3852794..3854059	UDP-N-acetylglucosamine 1-carboxyvinyltransferase
P0E69_18550	*murB*	4046461..4047498	UDP-N-acetylmuramate dehydrogenase
P0E69_16620	*murC*	3610420..3611895	UDP-N-acetylmuramate–L-alanine ligase
P0E69_16635	*murD*	3614246..3615562	UDP-N-acetylmuramoyl-L-alanine–D-glutamate ligase
P0E69_16650	*murE*	3617999..3619486	UDP-N-acetylmuramoyl-L-alanyl-D-glutamate–2,6-diaminopimelate ligase
P0E69_16645	*murF*	3616641..3618002	UDP-N-acetylmuramoyl-tripeptide–D-alanyl-D-alanine ligase
P0E69_16625	*murG*	3611917..3613023	undecaprenyldiphospho-muramoylpentapeptide beta-N-acetylglucosaminyltransferase
P0E69_19215	*murI*	4187076..4187939	glutamate racemase
P0E69_09125	*murJ*	2034271..2035806	murein biosynthesis integral membrane protein MurJ
P0E69_02530	*mpl*	533726..535093	UDP-N-acetylmuramate:L-alanyl-gamma-D-glutamyl-meso-diaminopimelate ligase
P0E69_10225	*mrdA, pbp2*	2245325..2247238	peptidoglycan D,D-transpeptidase MrdA; penicillin-binding protein 2
P0E69_12310	*mltG*	2694135..2695157	endolytic transglycosylase MltG
P0E69_14535	*mrdA*	3169626..3171530	peptidoglycan D,D-transpeptidase MrdA
P0E69_14540	*mrdB*	3171539..3172652	peptidoglycan glycosyltransferase MrdB
P0E69_16380	*mrcB*	3555552..3558161	bifunctional glycosyl transferase/transpeptidas
P0E69_16640	*mraY*	3615565..3616647	phospho-N-acetylmuramoyl-pentapeptide-transferase
P0E69_17650	*mtgA*	3833825..3834442	monofunctional biosynthetic peptidoglycan transglycosylase
P0E69_18200	*alr*	3963797..3964876	alanine racemase
P0E69_19885	*glmU*	4335096..4336466	bifunctional UDP-N-acetylglucosamine diphosphorylase/glucosamine-1-phosphate N-acetyltransferase GlmU
P0E69_19890	*glmS*	4336668..4338497	glutamine–fructose-6-phosphate transaminase (isomerizing)
P0E69_02635	*glmM*	551934..553271	phosphoglucosamine mutase
P0E69_04295	*uppS*	926815..927573	(2E,6E)-farnesyl-diphosphate-specific ditrans,polycis-undecaprenyl-diphosphate synthase

Another important factor contributing to the virulence of a bacterium is its flagellar motility. The movement of the flagella is guided by chemotaxis and aerotaxis receptors. Besides aiding in motility, the flagella also play a role in autoagglutination (Misawa and Blaser, 2000), adherence to epithelial cells, and the formation of biofilms (Haiko and Westerlund-Wikström, 2013). Additionally, a type III secretion system integrated into the flagellar apparatus allows for the secretion of various effector proteins (Dewoody et al., 2013). It is worth noting that the genes responsible for the production of flagellar proteins are located in the region 1,739,236..1,829,609 of the *C. arupi* DSM115072 genome, while the chemotaxis and aerotaxis receptors, along with their associated signal transduction proteins, are dispersed throughout the genome. Regarding a (flagellar) type III secretion system, there were no homologous genes found for the structural components and effector proteins secreted via a type III secretion system in any of the currently available representatives of the genus *Chimaeribacter*, as determined by a BLAST search for homologs of *yopH*, *yopE*, *yopT*, *ypkA*/*yopO*, *yopJ*, *yopM*, *yscT*, *yscS*, *yscQ*, *ssaD*, *yscE*, *yopR*/*yscH*, *yscF*, *yscG*, *yscI*, *yscJ*, and *yscL*.

Nearly all bacteria use two-component signal transduction systems that can process external signals to regulate the expression of a variety of genes, particularly those responsible for adaptation to specific environmental and habitat conditions, which includes some virulence-associated factors. Such two-component signal transduction systems typically consist of a sensor histidine kinase and a response regulator whose genes are usually located in immediate proximity in the bacterial genome. In the genome of *C. arupi* DSM115072, we detected 22 such two-component signal transduction systems whose predicted functions are listed in **Table 12**. Among the numerous two-component signal transduction systems, it is important to mention the PmrA/B system (P0E69_02595 & P0E69_02600) in relation to the pathogen’s virulence. This system has been shown to regulate the expression of the main virulence factor genes in *Erwinia carotovora* ssp. *carotovora* (Hyytiäinen et al., 2003). Additionally, in *P. aeruginosa*, it controls the genes involved in resistance to polymyxin B and cationic antimicrobial peptides (McPhee et al., 2003).

**Table 12:** Two-component signal transduction systems of *C. arupi* DSM115072.

Kinase	Response regulator	Position	Predicted function
P0E69_01085	P0E69_01080	259117.. 261236	two-component system response regulator OmpR & osmolarity sensory histidine kinase EnvZ
P0E69_02055	P0E69_02060	434878.. 437026	copper sensory histidine kinase CusS/silver sensor histidine kinase SilS & copper-sensing two-component system response regulator CusR/silver response regulator transcription factor SilR (copper homeostasis, cobalt-zinc-cadmium resistance)
P0E69_02595	P0E69_02600	543907.. 545627	two-component system sensor histidine kinase PmrB & two-component system response regulator PmrA
P0E69_04900	P0E69_04910	1046908.. 1050529	HAMP domain-containing sensor histidine kinase GlrK & transcriptional response regulatory protein GlrR
P0E69_05130	P0E69_05135	1091681.. 1093776	response regulator BaeR & sensory histidine kinase BaeS
P0E69_05355	P0E69_05350	1152348.. 1154831	two-component system response regulator NarL & nitrate/nitrite two-component system sensor histidine kinase NarQ
P0E69_05485	P0E69_05480	1182498.. 1184511	transcriptional regulatory protein CpxR & two-component system sensor histidine kinase CpxA; envelope stress response activation lipoprotein NlpE, an outer membrane lipoprotein, interacts directly with CpxA (P0E69_04420)
P0E69_05630	P0E69_05625	1208565.. 1211060	LytTR family DNA-binding domain-containing (YpdB) & protein & sensor histidine kinase LytS (YpdA)
P0E69_06175	P0E69_06180	1336245.. 1339907	sensor histidine kinase RcsC & DNA-binding capsular synthesis response regulator RcsB
P0E69_10050	P0E69_10055	2205123.. 2207146	sensory histidine kinase RstB & two-component transcriptional response regulator RstA
P0E69_12185	P0E69_12180	2665538.. 2667680	transcriptional regulatory protein PhoP & sensor histidine kinase PhoQ (lipid A modifications)
P0E69_13510	P0E69_13515	2955530.. 2957580	heavy metal sensor histidine kinase & DNA-binding heavy metal response regulator (cobalt-zinc-cadmium resistance)
P0E69_14315	P0E69_14320	3123599.. 3126972	osmosensitive K^+^ channel histidine kinase KdpD & DNA-binding response regulator KdpE
P0E69_15290	P0E69_15295	3327111.. 3329138	phosphate regulon sensor histidine kinase PhoR & phosphate response regulator transcription factor PhoB
P0E69_15920	P0E69_15925	3450426.. 3452793	sensor histidine kinase & response regulator
P0E69_16300	P0E69_16305	3533813.. 3537739	signal transduction histidine-protein kinase BarA & sugar diacid utilization regulator SdaR (D-galactarate, D-glucarate, and D-glycerate catabolism)
P0E69_16420	P0E69_16425	3565769.. 3567777	sensory histidine kinase QseC & two-component system response regulator QseB (quorum sensing) + two-component system QseEF-associated lipoprotein QseG (P0E69_04905)
P0E69_16975	P0E69_16980	3695107.. 3697316	sensor histidine kinase & response regulator
P0E69_17660	P0E69_17115	3835541.. 383788	aerobic respiration two-component sensor histidine kinase ArcB & two-component system response regulator ArcA (not close proximity)
P0E69_19420	P0E69_19415	4235105.. 4237170	copper-sensing two-component system response regulator CpxR & copper sensory histidine kinase CpxA envelope stress response activation lipoprotein NlpE, an outer membrane lipoprotein, interacts directly with CpxA (P0E69_04420)
P0E69_19690	P0E69_19695	4292446.. 4294916	nitrogen regulation protein NR(II)/NtrB/GlnL & nitrogen regulation protein NR(I)/GlnG/NtrC (controls expression of the nitrogen-regulated (ntr) genes in response to nitrogen limitation)
P0E69_20295	P0E69_20290	53081.. 55109	putative two-component system response regulator YedW.Putative two component system histidine kinase YedV/HprR (heavy metal)

The PhoP/Q (P0E69_12185 & P0E69_12180) two-component signal transduction system plays a crucial role in regulating genes that are necessary for the intracellular survival of *Salmonella typhimurium* and resistance to cationic peptides. Recent studies using mass spectrometry have shown that PhoP/Q controls the structural modifications of lipid A by adding aminoarabinose and 2-hydroxymyristate. These modified lipid A molecules have a significant impact on the expression of the adhesion molecule E-selectin in endothelial cells and the production of tumor necrosis factor-alpha in adherent monocytes (Guo et al., 1997). Additionally, PhoP/PhoQ induces the production of a lipase called PagL, which is responsible for the 3-*O*-deacylation of lipid A precursors in *Salmonella typhimurium*. This modification alters the charge and hydrophobicity of the lipid A molecule, influencing the bacterium’s interaction with host cells and the host immune system. The changes in lipid A mediated by PagL also affect signaling through Toll-like receptor 4, which is crucial for the recognition of bacterial pathogens by the host immune system (Trent et al., 2001; Kawasaki et al., 2004).

In the context of antibiotic resistance, it is important to mention the BaeR/S system. This system responds to envelope stress or spheroplast formation and contributes to resistance against novobiocin and bile salts by activating the expression of the drug exporter gene *mdtABC* (Nagakubo et al., 2002).

There are several situations where bacteria need to counteract the harmful effects of Reactive Oxygen Species (ROS). Especially, after being engulfed by host cells or immune cells through phagocytosis, bacteria must defend themselves against ROS encountered in the phagolysosome to ensure their survival inside the cell. In the genome of *C. arupi* DSM115072, 23 coding sequences (CDSs) were identified (**Table 13**) encoding gene products belonging to this specific functional category including e.g. two predicted superoxide dismutase genes, one presumptive catalase, three predicted thioredoxins, and two predicted thioredoxin reductases. In addition, we found two copies encoding the biofilm peroxide resistance protein BsmA (P0E69_02430 & P0E69_12140). This protein plays a role to resist acid and peroxide stress in biofilms and promotes microcolony formation, which precedes biofilm formation, by inhibiting flagellar motility (Weber et al., 2010).

**Table 13:** Antioxidant factors of *C. arupi* DSM115072.

Locus tag	Gene	Position	Predicted function
P0E69_00230	*sodA*	48539..49162	superoxide dismutase [Mn^2+^]
P0E69_00440	*ohrB*	95354..95779	organic hydroperoxide resistance protein
P0E69_02430	*bmsA*	512351..512659	biofilm peroxide resistance protein BsmA
P0E69_02500	*msrA*	524986..525621	peptide-methionine (S)-S-oxide reductase MsrA
P0E69_02540	*yhcN*	535610..535870	peroxide/acid stress response protein YhcN
P0E69_02545	*yhcN*	536006..536269	peroxide/acid stress response protein YhcN
P0E69_03070	*trx-GI*	660012..660452	heat resistance system thioredoxin Trx-GI
P0E69_05270	*bcp*	1133791..1134261	thioredoxin-dependent thiol peroxidase
P0E69_06695		1506872..1507075	thioredoxin reductase
P0E69_09415		2087216..2087419	thioredoxin reductase
P0E69_09865	*katE*	2152351..2154628	catalase HPII
P0E69_09915	*msrB*	2172804..2173217	peptide-methionine (R)-S-oxide reductase MsrB
P0E69_09975	*tpx*	2184435..2184938	thiol peroxidase
P0E69_11445	*sodC*	2519256..2519777	superoxide dismutase [Cu-Zn] SodC
P0E69_11665		2562846..2563397	glutathione peroxidase/thioredoxin peroxidase
P0E69_12140	*bsmA*	2656927..2657235	biofilm peroxide resistance protein BsmA
P0E69_13125	*trxB*	2861489..2862463	thioredoxin-disulfide reductase
P0E69_15240		3311777..3312379	peroxiredoxin C
P0E69_15980	*trxC*	3464961..3465389	thioredoxin TrxC
P0E69_17060	*yaaA*	3715309..3716082	peroxide stress protein YaaA
P0E69_19075	*trxA*	4156666..4156992	thioredoxin TrxA
P0E69_20270		47505..48302	carboxymuconolactone decarboxylase family protein
P0E69_21395		302447..302749	carboxymuconolactone decarboxylase family protein

## 5 Conclusion

In summary, *C. arupi* is a bacterium of the order Enterobactereales that has so far been detected quite rarely. The sources of the isolates available so far, the frequent detection of the *C. arupi* 16S rRNA gene in human gut metagenomes, along with the existence of genes encoding TcfC-like α-Pili, suggests that this bacterium is well-adapted to humans similar to *S. enterica* serotype Typhi and *S. enterica* serotype Paratyphi A. Therefore, it is likely that the human intestine serves as one habitat of the bacterium. *C. arupi* has genes encoding proteins to facilitate flagellar and type IV pili-mediated motility and a number of γ_1_-pili and σ-pili, as well as other factors that allow biofilm formation (e.g. on abiotic surfaces such as an implanted Broviac catheter), so it is likely that the bacterium moves in the chyme and the mucus of the intestine and forms mucosal biofilms on fiber particles. With these presumed flagella and pili, the bacterium should be capable of adhering to host epithelia, particularly the intestinal epithelium. It may then invasively breach the epithelial barrier, assisted by long polar fimbriae. Antioxidant factors may help it temporarily survive in phagolysosomes. In tissue, it may be protected from phagocytosis by a capsule and can access iron ions from erythrocytes via the type 6 secretion system and hemolysins. Furthermore, *C. arupi* possesses all the necessary genes for producing the complete set of enzymes required for the synthesis of LPS. LPS, a crucial constituent of the outer membrane in Gram-negative bacteria, often plays a pivotal role in disease development as it can strongly activate the immune system and potentially lead to sepsis. In the described case, the bacterium may have actively entered the patient’s bloodstream. Apart from beta-lactam mediated penicillin and aminopenicillin resistance, most antibiotic therapeutic options are open in the case of a *C. arupi* bloodstream infection. To better diagnose the germ in the future, it was sent to Bruker Daltonics to be added to the spectra database.

## Data availability statement

The datasets presented in this study can be found in online repositories. The names of the repository/repositories and accession number(s) can be found below: https://www.ncbi.nlm.nih.gov/genbank/, CP119395; https://www.ncbi.nlm.nih.gov/genbank/, CP119396; https://www.ncbi.nlm.nih.gov/genbank/, CP119397; https://www.ncbi.nlm.nih.gov/genbank/, CP119398.

## Ethics statement

Ethical approval was not required for the study involving humans in accordance with the local legislation and institutional requirements. Written informed consent to participate in this study was not required from the participants or the participants’ legal guardians/next of kin in accordance with the national legislation and the institutional requirements.

## Author contributions

MR: Conceptualization, Data curation, Formal Analysis, Investigation, Methodology, Software, Visualization, Writing – review & editing. KH: Data curation, Investigation, Writing – original draft, Writing – review & editing. RI: Investigation, Writing – original draft, Writing – review & editing. AD: Data curation, Software, Visualization, Writing – review & editing. AT: Data curation, Formal Analysis, Software, Writing – review & editing. PM: Project administration, Software, Writing – review & editing. AK: Funding acquisition, Resources, Writing – review & editing. AZ: Conceptualization, Investigation, Methodology, Project administration, Software, Supervision, Writing – original draft, Writing – review & editing.
