# Supplementary material for: Chimaeribacter arupi a new member of the Yersineacea family has the characteristics of a human pathogen
Source: Front Cell Infect Microbiol. 2023 Oct 6;13:1277522. doi: 10.3389/fcimb.2023.1277522 (PMC10587679; doi:10.3389/fcimb.2023.1277522)
Supplement: Supplementary file 1 [file DataSheet_1.pdf]

Supplementary Table 1: Biochemical test results (API 50 CH, API 20 E, Vitek2 GN card)

	characteristic	*C. arupi* DSM115072	*C. arupi* 2016-Iso1	*C. arupi* 2016-Iso2	*C. arupi* 2016-Iso3	*C. arupi* 2013-Iso5
API50CH	fermentation of:					
	Glycerol	(+)	+	-	-	+
	Erythriol	-	-	-	-	-
	D-Arabinose	(+)	-	-	-	+
	L-Arabinose	+	+	-	+	+
	D-Ribose	+	+	+	+	+
	D-Xylose	+	+	+	+	+
	L-Xylose	-	-	-	-	-
	D-Adonitol	-	-	-	-	-
	Methyl-β-D-xylopyranoside	-	-	-	-	-
	D-Galactose	+	+	+	+	+
	D-Glucose	+[c v]	+	+	+	+
	D-Fructose	+	+	+	+	+
	D-Mannose	+[v]	+	+	+	+
	L-Sorbose	-	-	-	-	-
	L-Rhamnose	+[c]	+	+	+	+
	Dulcitol	+	+	-	+	+
	Inositol	+[c]	+	+	+	+
	D-Mannitol	+[c v]	+	+	+	+
	D-Sorbitol	+[c v]	+	+	+	+
	Methyl-α-D-mannopyranoside	-	-	-	-	-
	Methyl-α-D-glucopyranoside	+	+	+	+	+
	N-Acetyl-Glucosamine	+[v]	+	+	+	+
	Amygdalin	-/+[d]	-	-	+	+
	Arbutin	+	+	+	+	+
	Aesculin ferric citrate	+	+	+	+	+
	Salicin	+	+	+	+	+
	D-Cellobiose	+[v]	+	+	+	+
	D-Maltose	+[v]	+	+	+	+
	D-Lactose	(+)	+	+	+	+
	D-Melibiose	+[c]	+	+	+	+
	Sucrose	-[c v]	-	+	+	+
	D-Trehalose	+[v]	+	+	+	+
	Inulin	-	-	-	-	-
	D-Melezitose	-	-	-	-	-

	D-Raffinose	(+)	+	+	+	+
	Starch (Amidon)	(+)	-	-	-	-
	Glycogen	-	-	-	-	-
	Xylitol	-	-	-	-	-
	Gentiobiose	+	+	+	+	+
	D-Turanose	+	+	+	+	+
	D-Lyxose	-	-	-	-	-
	D-Tagatose	- [v]	-	-	-	-
	D-Fucose	-	-	-	-	-
	L-Fucose	-	-	-	-	-
	D-Arabitol	-	-	-	-	-
	L-Arabitol	- [v]	-	-	-	-
	Potassium-Gluconate	-	+	-	-	-
	Potassium-2-ketogluconate	(+)	+	+	+	+
	Potassium-5-ketogluconate	- [v]	-	-	-	-
API 20E						
	β-Galactosidase (ONPG)	-/+[w]	-	-	-	-
	Arginine dihydrolase	-	-	-	-	-
	Lysine decarboxylase	- [v]	-	-	-	-
	Ornithine decarboxylase	- [v]	-	-	-	-
	Citrate utilization	(+)/+[w]	+	+	+	+
	H2S production	-	-	-	-	-
	Urease	-[v]	-	-	-	-
	Tryptophan deaminase	-	-	-	-	-
	Indole production	-	-	-	-	-
	Acetoin production (Voges–Proskauer test)	-	-	-	-	-
	Gelatinase	(+)	+	+	+	-
	Oxidase	-	-	-	-	-
Vitek2 GN card						
	Ala-Phe-Pro-ARYLAMIDASE	-				
	L-Pyrrolydonyl-ARYLAMIDASE	+				
	Glutamyl-Arylamidase-pNA	-				
	GAMMA-GLUTAMYL-TRANSFERASE	-				
	BETA-GLUCOSIDASE	+				
	BETA-XYLOSIDASE	-				

	BETA-Alanin-Arylamidase-pNA	-				
	L-Prolin-ARYLAMIDASE	-				
	LIPASE	-				
	PALATINOSE	-				
	Tyrosin-ARYLAMIDASE	-				
	MALONAT	-				
	L-LAKTAT- alkalisation	-				
	ALPHA-GLUCOSIDASE	-				
	SUCCINAT- alkalisation	-				
	Beta-N-ACETYL-GALACTOSAMINIDASE	+				
	PHOSPHATASE	-				
	Glycin-ARYLAMIDASE	-				
	L-HISTIDIN-assimilation	-				
	COUMARAT	-				
	BETA-GLUCURONIDASE	-				
	O/129- resistance (comp.vibrio.)	+				
	Glu-Gly-Arg-ARYLAMIDASE	-				
	L-MALAT-assimilation	-				
	ELLMAN	-				
	L-LACTAT-assimilation	-				

c) concordant results in API50CH and API20E
d) divergent results in API50CH and API20E
v) concordant results in API50CH/API20E and Vitek2
w) divergent results in API50CH/API20E and Vitek2

Supplementary Table 2: List of coding sequences representing an incomplete phage locus on the chromosome of *C. arupi* DSM115072

No.	CDS Position	orientation	Locus Tag	BLAST Hit	E-value
1	1001607..1001632	+	-	attL	0.0
2	1002749..1005064	-	P0E69_04675	PHAGE_Entero_P4_NC_001609: DNA primase; PP_00972; phage(gi9627512)	0.0
3	1005045..1005419	-	P0E69_04680	PHAGE_Entero_P4_NC_001609: hypothetical protein; PP_00973; phage(gi9627513)	7.33e-19
4	1005409..1005675	-	P0E69_04685	hypothetical; PP_00974	0.0
5	1005672..1005866	-	P0E69_04690	PHAGE_Entero_P4_NC_001609: putative CI repressor; PP_00975; phage(gi9627516)	3.91e-22
6	1006213..1006461	-	P0E69_04695	hypothetical; PP_00976	0.0
7	1006458..1006715	-	P0E69_04700	PHAGE_Entero_P4_NC_001609: transcriptional regulator; PP_00977; phage(gi9627517)	3.44e-26
8	1007239..1007955	+	P0E69_04705	PHAGE_Entero_P4_NC_001609: head size determination protein sid; PP_00978; phage(gi9627518)	5.73e-18
9	1008308..1008475	+	P0E69_04715	hypothetical; PP_00979	0.0
10	1008544..1009491	+	P0E69_04720	hypothetical; PP_00980	0.0
11	1009501..1012149	+	P0E69_04725	PHAGE_Acidia_6_NC_010152: putative helicase; PP_00981; phage(gi162329366)	4.56e-10
12	1012146..1013138	-	P0E69_04730	PHAGE_Entero_P4_NC_001609: integrase; PP_00982; phage(gi9627511)	2.58e-60
13	1013244..1013357	-	P0E69_04730	PROPHAGE_Escher_MG1655: CP4-57 prophage; integrase; PP_00983; phage(gi16130540)	1.85e-08
14	1013406..1013431	+	-	attR	0.0
15	1013551..1013915	-	P0E69_04735	tRNA	0.0
16	1013965..1014447	-	P0E69_04740	PHAGE_Escher_500465_1_NC_049342: HNH endonuclease; PP_00984; phage(gi100046)	8.39e-99
17	1014459..1014596	-	-	hypothetical; PP_00985	0.0
18	1014745..1015041	+	P0E69_04745	PHAGE_Escher_500465_1_NC_049342: primase; PP_00986; phage(gi100047)	4.02e-54
19	1015034..1015318	+	P0E69_04750	PHAGE_Escher_500465_1_NC_049342: exonuclease; PP_00987; phage(gi100048)	1.67e-43
20	1015386..1015733	-	P0E69_04755	PHAGE_Escher_500465_1_NC_049342: recombination protein; PP_00988; phage(gi100049)	9.71e-63
21	1015846..1017507	-	P0E69_04760	PHAGE_Escher_500465_1_NC_049342: putative single-stranded DNA binding protein; PP_00989; phage(gi100050)	0.0
22	1017594..1018472	-	P0E69_04765	PHAGE_Escher_500465_1_NC_049342: hypothetical protein; PP_00990; phage(gi100051)	4.64e-168
23	1018596..1019183	+	P0E69_04770	PHAGE_Escher_500465_1_NC_049342: tail assembly protein; PP_00991; phage(gi100053)	2.19e-40
24	1019236..1019925	-	P0E69_04775	PHAGE_Flavob_Fpv1_NC_031914: hypothetical protein; PP_00992; phage(gi100059)	1.73e-37
25	1019913..1020092	+	-	hypothetical; PP_00993	0.0
26	1020266..1020649	+	P0E69_04780	PHAGE_Klebsi_vB_KpnM_KpV477_NC_031087: DNA polymerase III alpha subunit; PP_00994; phage(gi100121)	1.09e-44
27	1020714..1022036	-	P0E69_04785	PHAGE_Acinet_vB_AbaM_ME3_NC_041884: deoxynucleoside kinase; PP_00995; phage(gi100082)	1.23e-09
28	1022232..1022948	+	P0E69_04790	hypothetical; PP_00996	0.0
29	1022923..1024578	-	P0E69_04795	hypothetical; PP_00997	0.0
30	1024718..1025368	+	P0E69_04800	PHAGE_Rhizob_vB_RleM_P10VF_NC_025429: RNA polymerase sigma-70 factor; PP_00998; phage(gi712913000)	2.96e-07

Supplementary Table 3: List of coding sequences representing the putative intact phage locus on the chromosome of *C. arupi* DSM115072

No.	CDS Position	orientation	Locus Tag	BLAST Hit	E-value
1	1500941..1500953	+	-	attL	0.0
2	1501032..1502348	+	P0E69_06650	PHAGE_Entero_HK140_NC_019710: integrase; PP_01410; phage(gi428781968)	0.0
3	1502329..1502520	+	P0E69_06655	AlpA family phage regulatory protein	
4	1502624..1502920	-	P0E69_06660	PHAGE_Xantho_CP2_NC_020205: hypothetical protein; PP_01411; phage(gi448245209)	1.57e-28
5	1504763..1505326	-	P0E69_06670	hypothetical; PP_01412	0.0
6	1505344..1505685	-	P0E69_06675	hypothetical; PP_01413	0.0
7	1505705..1506109	-	P0E69_06680	PHAGE_Salmon_vB_SosS_Oslo_NC_018279: hypothetical protein; PP_01414; phage(gi399528799)	6.41e-60
8	1506109..1506729	-	P0E69_06685	PHAGE_Salmon_vB_SosS_Oslo_NC_018279: essential recombination function Erf; PP_01415; phage(gi399528800)	7.12e-95
9	1506726..1506875	-	P0E69_06690	PHAGE_Salmon_vB_SosS_Oslo_NC_018279: Arf protein; PP_01416; phage(gi399528801)	5.21e-07
10	1506872..1507075	-	P0E69_06695	PHAGE_Erwini_vB_EhrS_49_NC_048197: hypothetical protein; PP_01417; phage(gi100043)	8.11e-08
11	1507072..1507200	+	P0E69_06700	hypothetical protein	
12	1507197..1507328	+	P0E69_06705	protease FtsH-inhibitory lysogeny factor CIII	
13	1507361..1508329	-	P0E69_06710	PHAGE_Entero_c_1_NC_019706: hypothetical protein; PP_01418; phage(gi428781776)	3.29e-101
14	1508441..1508572	-	P0E69_06715	hypothetical; PP_01419	0.0
15	1508592..1508780	-	P0E69_06720	PHAGE_Salmon_Lumpael_NC_048113: tail tip protein; PP_01420; phage(gi100054)	6.83e-14
16	1509158..1509343	-	P0E69_06730	PHAGE_Erwini_vB_EhrS_59_NC_048198: putative single-stranded DNA binding protein; PP_01421; phage(gi100050)	3.20e-07
17	1510175..1510438	-	P0E69_06735	PHAGE_Erwini_vB_EhrS_59_NC_048198: tail tip protein; PP_01422; phage(gi100054)	1.98e-24
18	1510907..1511125	+	P0E69_06740	PHAGE_Entero_Sf101_NC_027398: Cro; PP_01423; phage(gi849122302)	5.77e-27
19	1511400..1511522	+	P0E69_06745	PHAGE_Entero_mEpX2_NC_019705: CII protein; PP_01424; phage(gi428765658)	1.71e-13
20	1511564..1511704	+	P0E69_06750	hypothetical; PP_01425	0.0
21	1512010..1512600	+	P0E69_06755	PHAGE_Entero_HK022_NC_002166: gene O protein; PP_01426; phage(gi9634150)	9.03e-51
22	1512590..1514029	+	P0E69_06760	PHAGE_Entero_VT2_Sakai_NC_000902: similar to P protein of bacteriophage HK022; PP_01427; phage(gi9633423)	0.0
23	1514029..1514175	+	P0E69_06765	hypothetical; PP_01428	0.0
24	1514675..1514800	+	P0E69_06775	hypothetical; PP_01429	0.0
25	1514797..1515387	+	P0E69_06780	PHAGE_Erwini_vB_EhrS_59_NC_048198: hypothetical protein; PP_01430; phage(gi100065)	1.84e-131
26	1515384..1515602	+	P0E69_06785	protein NinH	
27	1515587..1515754	+	P0E69_06790	NinE family protein	
28	1515751..1516122	+	P0E69_06795	PHAGE_Pectob_phiTE_NC_020201: hypothetical protein; PP_01431; phage(gi448244760)	3.47e-15
29	1516106..1516249	+	P0E69_06800	PHAGE_Erwini_vB_EhrS_49_NC_048197: portal protein; PP_01432; phage(gi100069)	1.06e-05
30	1516239..1516439	+	P0E69_06805	PHAGE_Mycoba_Rebeuca_NC_042341: holin; PP_01433; phage(gi100034)	2.27e-07
31	1516929..1517081	+	P0E69_06815	PHAGE_Salmon_118970_sal4_NC_030919: holin; PP_01434; phage(gi100034)	2.51e-10
32	1517180..1517845	+	P0E69_06820	PHAGE_Erwini_vB_EhrS_59_NC_048198: hypothetical protein; PP_01435; phage(gi100073)	8.50e-111
33	1518784..1519122	+	P0E69_06825	hypothetical; PP_01436	0.0

34	1519196..1519417	+	P0E69_06830	hypothetical; PP_01437	0.0
35	1519699..1519881	+	P0E69_06835	PHAGE_Aeromo_LAh_8_NC_048775: hypothetical protein; PP_01438; phage(gi100112)	3.80e-15
36	1519878..1520222	+	P0E69_06840	PHAGE_Pseudo_vB_Pae_Kakheti25_NC_017864: holin; PP_01439; phage(gi388542661)	2.24e-12
37	1520219..1520506	+	P0E69_06845	hypothetical; PP_01440	0.0
38	1520753..1520932	+	P0E69_06850	hypothetical; PP_01441	0.0
39	1520944..1521183	+	P0E69_06855	hypothetical; PP_01442	0.0
40	1521438..1521782	+	P0E69_06860	PHAGE_Pseudo_phi_2_NC_013638: hypothetical protein; PP_01443; phage(gi281306701)	4.28e-05
41	1521791..1521961	+	P0E69_06865	hypothetical; PP_01444	0.0
42	1522015..1522458	+	P0E69_06870	PHAGE_Pseudo_vB_PaeP_Tr60_Ab31_NC_023575: Putative terminase small subunit; PP_01445; phage(gi589286873)	7.26e-50
43	1522523..1523761	+	P0E69_06875	PHAGE_Pelagi_HTVC010P_NC_020481: phage terminase large subunit; PP_01446; phage(gi460042259)	1.82e-85
44	1523821..1523949	+	P0E69_06880	hypothetical; PP_01447	0.0
45	1524013..1525665	+	P0E69_06885	PHAGE_Pelagi_HTVC010P_NC_020481: head-tail connector protein; PP_01448; phage(gi460042262)	1.86e-85
46	1525790..1525996	+	P0E69_06890	PHAGE_Burkho_vB_BmuP_KL4_NC_047958: hypothetical protein; PP_01449; phage(gi100063)	1.06e-09
47	1526041..1526172	-	-	hypothetical; PP_01450	0.0
48	1526220..1526699	+	P0E69_06895	PHAGE_Pseudo_vB_PaeP_Tr60_Ab31_NC_023575: Putative endoprotease; PP_01451; phage(gi589286878)	7.15e-11
49	1526841..1527719	+	P0E69_06900	PHAGE_Pseudo_vB_PaeP_Tr60_Ab31_NC_023575: Putative major capsid protein; PP_01452; phage(gi589286879)	1.51e-156
50	1527729..1528175	+	P0E69_06905	PHAGE_Pseudo_vB_PaeP_Tr60_Ab31_NC_023575: Hypothetical Protein; PP_01453; phage(gi589286880)	8.44e-54
51	1528212..1528808	+	P0E69_06910	PHAGE_Pseudo_vB_PaeP_Tr60_Ab31_NC_023575: Putative tail tubular protein A; PP_01454; phage(gi589286882)	1.39e-18
52	1528809..1531085	+	P0E69_06915	PHAGE_Burkho_vB_BmuP_KL4_NC_047958: minor tail protein; PP_01455; phage(gi100055)	2.98e-151
53	1531055..1531273	-	-	hypothetical; PP_01456	0.0
54	1531274..1531498	+	P0E69_06920	hypothetical; PP_01457	0.0
55	1531971..1534592	+	P0E69_06930	PHAGE_Pseudo_vB_PaeP_Tr60_Ab31_NC_023575: Putative structural lysozyme; PP_01458; phage(gi589286886)	1.12e-32
56	1545284..1545296	+	-	attR	0.0

Supplementary Table 4: List of coding sequences representing a questionable phage locus on the chromosome of *C. arupi* DSM115072

No.	CDS Position	orientation	Locus Tag	BLAST Hit	E-value
1	2081008..2081030	+		attL	0.0
2	2081064..2082356	-	P0E69_09345	PHAGE_Salmon_Fels_1_NC_010391: putative bacteriophage integrase; PP_01978; phage(gi169257156)	7.18e-174
3	2082387..2082644	-	P0E69_09350	PHAGE_Phage_Gifsy_2_NC_010393: bacteriophage excisionase; PP_01979; phage(gi169257269)	2.15e-33
4	2082899..2083138	+	P0E69_09355	hypothetical; PP_01980; DUF551 domain-containing protein	0.0
5	2083135..2083368	+	P0E69_09360	Hypothetical; DUF4060 family protein	
6	2083352..2083630	-	P0E69_09365	PHAGE_Erwini_phiEt88_NC_015295: hypothetical protein; PP_01981; phage(gi327198595)	1.03e-29
7	2084247..2084831	-	P0E69_09380	hypothetical; PP_01982	0.0
8	2084835..2085104	-	P0E69_09385	PHAGE_Salmon_35_NC_048632: hypothetical protein; PP_01983; phage(gi100058)	5.23e-18
9	2085988..2086380	-	P0E69_09400	PHAGE_Escher_Sortsne_NC_048178: hypothetical protein; PP_01984; phage(gi100025)	1.66e-60
10	2086450..2087073	-	P0E69_09405	PHAGE_Erwini_vB_EhrS_49_NC_048197: DNA adenine methyltransferase; PP_01985; phage(gi100041)	2.32e-100
11	2087070..2087219	-	P0E69_09410	PHAGE_Salmon_vB_SosS_Oslo_NC_018279: Arf protein; PP_01986; phage(gi399528801)	5.21e-07
12	2087216..2087368	-	P0E69_09415	hypothetical; PP_01987; thioredoxin reductase	0.0
13	2087544..2087672	-	P0E69_09425	protease FtsH-inhibitory lysogeny factor CIII	
14	2089004..2089804	-	P0E69_09450	PHAGE_Erwini_vB_EhrS_59_NC_048198: tail length tape-measure protein; PP_01988; phage(gi100057)	3.03e-127
15	2089871..2090053	-	P0E69_09455	hypothetical; PP_01989	0.0
16	2090917..2091207	-	P0E69_09465	PHAGE_Cronob_ENT47670_NC_019927: putative transcriptional regulator; PP_01990; phage(gi431810510)	5.46e-62
17	2092530..2093378	+	P0E69_09480	PHAGE_Entero_SfI_NC_027339: replication protein O; PP_01991; phage(gi849250271)	1.67e-118
18	2093375..2094070	+	P0E69_09485	PHAGE_Erwini_vB_EhrS_59_NC_048198: hypothetical protein; PP_01992; phage(gi100059)	9.35e-88
19	2094306..2094476	+	P0E69_09495	hypothetical; PP_01993	0.0
20	2095538..2095981	+	P0E69_09510	YbcN family protein	
21	2095978..2096193	+	P0E69_09515	protein NinH	
22	2096178..2096345	+	P0E69_09520	NinE family protein	
23	2096694..2096834	+	P0E69_09530	PHAGE_Erwini_vB_EhrS_49_NC_048197: portal protein; PP_01994; phage(gi100069)	4.88e-06
24	2096827..2097459	+	P0E69_09535	recombination protein NinG	
25	2097981..2098097	+	P0E69_09540	hypothetical; PP_01995; HNH endonuclease signature motif containing protein	0.0
26	2098094..2098705	+	P0E69_09545	PHAGE_Cronob_ENT47670_NC_019927: hypothetical protein; PP_01996; phage(gi431810516)	9.71e-48
27	2099060..2099179	-	-	hypothetical; PP_01997	0.0
28	2099577..2099987	-	P0E69_09550	type II toxin-antitoxin system HicB family antitoxin	
29	2100050..2100238	-	P0E69_09555	type II toxin-antitoxin system HicA family toxin	
30	2100454..2100804	-	P0E69_09560	phage holin, lambda family	
31	2100791..2101423	+	P0E69_09565	PHAGE_Cronob_ENT47670_NC_019927: putative endolysin; PP_01998; phage(gi431810515)	2.11e-64

32	2101411..2101866	+	P0E69_09570	Rz lytic protein	
33	2102579..2102728	+	0E69_09580	YnfU family zinc-binding protein	
34	2102938..2103615	+	P0E69_09585	PHAGE_Salmon_118970_sal4_NC_030919: hypothetical protein; PP_01999; phage(gi100042); Rha family transcriptional regulator	6.24e-136
35	2103734..2104453	+	P0E69_09590	GIY-YIG nuclease family protein	
36	2104515..2104961	+	P0E69_09595	ubiquitin carboxyl-hydrolase	
37	2104945..2106240	+	P0E69_09600	PHAGE_Entero_ES18_NC_006949: gp2; PP_02000; phage(gi62362215); phage terminase large subunit	0.0
38	2106251..2107684	+	P0E69_09605	PHAGE_Cronob_ENT47670_NC_019927: hypothetical protein; PP_02001; phage(gi431810500)	0.0
39	2107722..2108609	+	P0E69_09610	phage head morphogenesis protein	
40	2108199..2108324	-	-	hypothetical; PP_02002	0.0
41	2108635..2108907	+	P0E69_09615	hypothetical protein	
42	2108960..2110147	+	P0E69_09620	PHAGE_Erwini_vB_EhrS_59_NC_048198: hypothetical protein; PP_02003; phage(gi100005)	6.16e-85
43	2110151..2110582	+	P0E69_09625	PHAGE_Cronob_ENT47670_NC_019927: hypothetical protein; PP_02004; phage(gi431810525)	5.70e-68
44	2110594..2111661	+	P0E69_09630	PHAGE_Cronob_ENT47670_NC_019927: hypothetical protein; PP_02005; phage(gi431810503)	0.0
45	2111672..2112031	+	P0E69_09635	PHAGE_Erwini_vB_EhrS_59_NC_048198: hypothetical protein; PP_02006; phage(gi100008)	1.85e-36
46	2112667..2112930	+	P0E69_09650	PHAGE_Salmon_IME207_NC_031924: tail length tape-measure protein; PP_02007; phage(gi100057)	1.79e-18
47	2112932..2113300	+	P0E69_09655	PHAGE_Cronob_ENT47670_NC_019927: hypothetical protein; PP_02008; phage(gi431810532); HK97 gp10 family phage protein	3.96e-58
48	2114210..2114953	+	P0E69_09670	PHAGE_Cronob_ENT47670_NC_019927: major tail subunit; PP_02009; phage(gi431810520)	2.53e-103
49	2115440..2115679	+	P0E69_09675	PHAGE_Cronob_ENT47670_NC_019927: hypothetical protein; PP_02010; phage(gi431810508)	8.32e-29
50	2115944..2116102	+	P0E69_09680	hypothetical; PP_02011	0.0
51	2116121..2117287	+	P0E69_09685	hypothetical; PP_02012	0.0
52	2117380..2120772	+	P0E69_09690	PHAGE_Vibrio_pYD38_A_NC_021534: tail length tape measure protein; PP_02013; phage(gi514051037)	0.0
53	2132473..2132495	+	-	attR	0.0

Supplementary Table 5: List of coding sequences representing an incomplete phage locus on plasmid p353_DSM115072

No.	CDS Position	orientation	Locus Tag	BLAST Hit	E-value
1	223455..223877	-	P0E69_21070	PHAGE_Entero_SfV_NC_003444: putative flippase; PP_00223; phage(gi19549013)	3.84e-39
2	224053..224172	-	-	hypothetical; PP_00224	0.0
3	224401..225942	+	P0E69_21075	hypothetical; PP_00225, methyl-accepting chemotaxis protein	0.0
4	226022..226453	-	P0E69_21080	hypothetical; PP_00226, DUF2000 domain-containing protein	0.0
5	226594..227412	+	P0E69_21085	PHAGE_Bordet_vB_BbrM_PHB04_NC_047861: exonuclease; PP_00227; phage(gi100048); AraC family transcriptional regulator	3.18e-13
6	227434..228165	-	P0E69_21090	PHAGE_Ralsto_RSY1_NC_025115: hypothetical protein; PP_00228; phage(gi691326853)	2.52e-05
7	228165..228899	-	P0E69_21095	PHAGE_Ralsto_RSY1_NC_025115: hypothetical protein; PP_00229; phage(gi691326853)	2.76e-05
8	228899..229615	-	P0E69_21100	PHAGE_Ralsto_RSY1_NC_025115: hypothetical protein; PP_00230; phage(gi691326853)	1.88e-06
9	229612..231888	-	P0E69_21105	PHAGE_Ralsto_RSY1_NC_025115: hypothetical protein; PP_00231; phage(gi691326855)	4.41e-34
10	231885..234443	-	P0E69_21110	PHAGE_Ralsto_RSY1_NC_025115: hypothetical protein; PP_00232; phage(gi691326852)	1.82e-13
